# Trajectory and Functional Analysis of PD‐1^high^ CD4^+^CD8^+^ T Cells in Hepatocellular Carcinoma by Single‐Cell Cytometry and Transcriptome Sequencing

**DOI:** 10.1002/advs.202000224

**Published:** 2020-05-18

**Authors:** Bo Zheng, Dongfang Wang, Xinyao Qiu, Guijuan Luo, Tong Wu, Shuai Yang, Zhixuan Li, Yanjing Zhu, Shan Wang, Rui Wu, Chengjun Sui, Ziqi Gu, Siyun Shen, Seogsong Jeong, Xuan Wu, Jin Gu, Hongyang Wang, Lei Chen

**Affiliations:** ^1^ National Center for Liver Cancer Shanghai 200438 China; ^2^ The International Cooperation Laboratory on Signal Transduction Eastern Hepatobiliary Surgery Hospital Second Military Medical University Shanghai 200438 China; ^3^ MOE Key Laboratory for Bioinformatics BNRIST Bioinformatics Division Department of Automation Tsinghua University Beijing 100084 China; ^4^ Fudan University Shanghai Cancer Center Department of Oncology Shanghai Medical College Fudan University Shanghai 200032 China; ^5^ Department of Biliary Surgery I Eastern Hepatobiliary Surgery Hospital Second Military Medical University Changhai Road 225 Shanghai 200438 China; ^6^ Department of Liver Surgery Renji Hospital School of Medicine Shanghai JiaoTong University Shanghai 200127 China; ^7^ Department of Laboratory Medicine The Tenth People's Hospital of Shanghai Tongji University Shanghai 200072 China

**Keywords:** double positive T cells, liver cancer, mass cytometry, single‐cell sequencing, tumor microenvironment

## Abstract

The spatial heterogeneity of immune microenvironment in hepatocellular carcinoma (HCC) remains elusive. Here, a single‐cell study involving 17 432 600 immune cells of 39 matched HCC (T), nontumor (N), and leading‐edge (L) specimens by mass cytometry is conducted. The tumor‐associated CD4/CD8 double‐positive T (DPT) cells are found enriched in L regions with synergetic expression of PD‐1/HLA‐DR/ICOS/CD45RO and exhibit a higher level of IFN‐*γ*, TNF‐*α*, and PD‐1 upon stimulation. The enrichment of DPT and PD‐1^+^DPT in L regions indicates favorable prognosis. These tumor‐associated DPT cells with similar phenotype are also verified in other tumors and HCC animal models. Single‐cell RNA‐seq further characterizes the molecular features of DPT cells and uncovers 11 clusters with different cytotoxicity, exhaustion, and activation scores. TCR‐based trajectory analysis reveals that tumor‐associated DPT clusters share separated ancestries with local CD4^+^ or CD8^+^SPT cells rather than CD3^+^PBMC cells. TCR clones with frequency above 10 are mainly found coexisting in DPT and CD8^+^SPT cells. Specifically, PD‐1^high^DPT cluster (TDPT_10) shares the same ancestry with exhausted CD8^+^SPT cluster (TCD8T_2) and shows higher expression similarity and closer pathological location to PD‐1^+^CD8^+^ than PD‐1^+^CD4^+^T cells. Together, this study systematically characterizes the unique distribution of PD‐1^+^DPTs in HCC and puts forward new insights for the function and origin of tumor‐associated DPT cells.

## Graphical abstract

A time‐of‐flight mass cytometry‐based single‐cell proteomic study is conducted to explore the immune heterogeneity in hepatocellular carcinoma. The tumor‐associated double‐positive T (DPT) cells are found enriched in L regions with expression of PD‐1/HLA‐DR/ICOS/CD45RO. Single‐cell RNA‐seq uncovers the 11 subclusters with different cytotoxicity, exhaustion, and activation. TCR‐seq reveals DPT cells share separated ancestries with local CD4^+^ or CD8^+^SPT cells rather than CD3^+^PBMC cells.

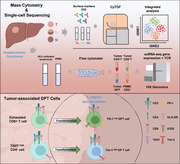

## 1 Introduction

Cancer is one of the most deadly diseases worldwide.^[^ ^1^ ^]^ In recent years, immunotherapy is regarded as a very promising therapeutic strategy for cancer, focusing on reactivating the immunosuppressive environment induced by cancer cells and improving antitumor immune responses.^[^ ^2^ ^]^ However, immunotherapy still faces many challenges. Even for the most popular anti‐PD‐1/PD‐L1 and anti‐CTLA4 therapy, only a small group of people show durable response, suggesting that a more in‐depth investigation of cancer immunity is needed.^[^ ^3^ ^]^

Spatial heterogeneity of immune microenvironment in tumors is the major challenge for cancer immunotherapy and has drawn wide attention.^[^ ^2^ ^]^ The crosstalks between tumor cells and infiltrated immune cells orchestrate every stage of tumorigenesis.^[^ ^4^ ^]^ It has been reported that spatial immune heterogeneity exists in lung cancer,^[^ ^5^ ^]^ breast cancer,^[^ ^6^ ^]^ and several other cancers, of which hepatocellular carcinoma (HCC) was regarded highly heterogeneous owing to the joint contributions of epigenetic alterations, virus infection, alcohol intake, and metabolic disorder during carcinogenesis. HCC microenvironment displays a more complicated and immunosuppressive phenotype than nontumor tissue which induces tumor escape and facilitates disease progression.^[^ ^7^, ^8^ ^]^ However, the transition process where the immune microenvironments goes from activated to suppressed condition remains unclear. As a “transition” zone, the tumor leading edge regions need to gain more attention. For example, it has been reported that PD‐1^+^B cells, enriched in L regions, can promote HCC progression through IL‐10 signaling.^[^ ^9^ ^]^ Neutrophils residing in L regions can accelerate tumor progression by inducing angiogenesis.^[^ ^10^ ^]^

In our study, we collected 39 matched HCC (T), nontumor (N), and leading‐edge (L) specimens from 13 HCC patients. By using single‐cell‐scaled time‐of‐flight mass cytometry (CyTOF), it was found that the immune cell compositions showed spatial changes from N to L to T regions. By deep analysis of T cell populations, we identified the L region displayed a unique T cell composition. Especially, we found that a small subpopulation of CD4/CD8 double positive T cells (DPT) coexpressing PD‐1/HLA‐DR/ICOS/CD45RO were enriched in the L region and have an activated phenotype. DPT is a group of well‐described premature T cell mainly existing in thymus.^[^ ^11^ ^]^ Our study identified the existence of tumor‐associated DPT cells in HCC with robust functionality. The existence of PD‐1^+^DPT cells was also confirmed by multiplex immunofluorescence tissue staining and its enrichment showed favorable prognostic value. Single‐cell RNA‐seq and TCR‐seq further characterized the molecular features and clonotypes of DPT cells. Results suggest that tumor‐associated DPT cells may derive from infiltrating CD4^+^ or CD8^+^ single positive T (SPT) cells.

## 2 Results

### 2.1 The Spatial Heterogeneities of Major Immune Lineages in Hepatocellular Carcinoma

**Figure 1:**
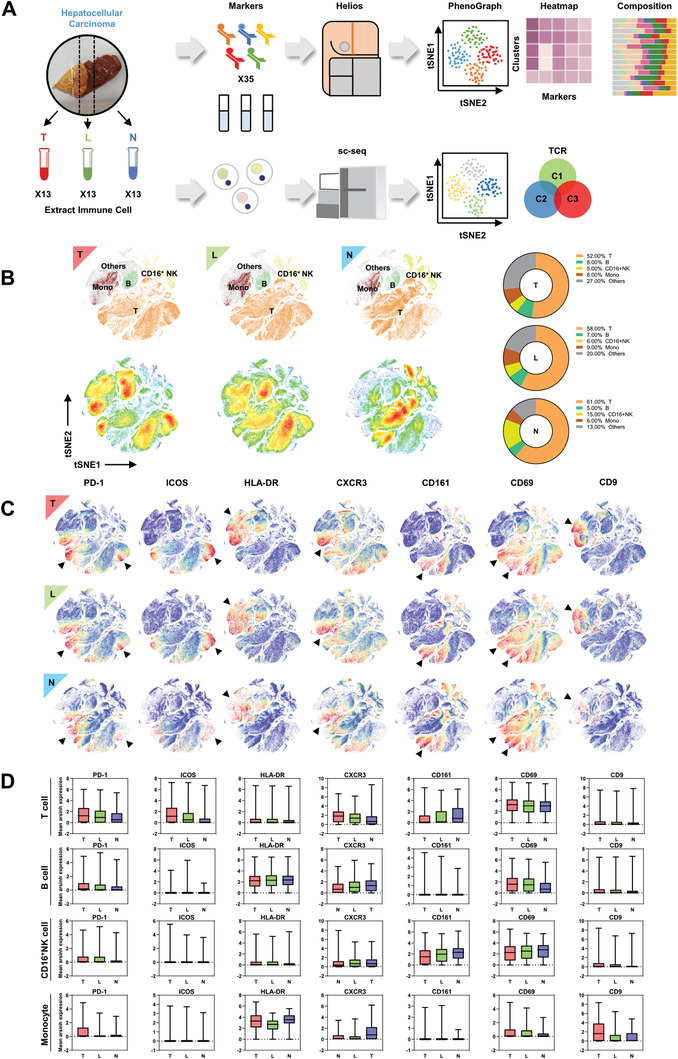
The spatial heterogeneities of major immune cell lineages in hepatocellular carcinoma. A) Graphical abstract of the whole workflow. Surgical resected samples were extracted for immune cells and processed with metal‐labeled antibodies and put into time‐of‐flight mass cytometry pipeline. Acquired data were visualized after dimension reduction. Cell clusters were identified by manual gating strategy and clustering algorithm. T refers to tumor core region; L refers to leading‐edge area; N refers to nontumor region. B) tSNE plots of CyTOF data from tumor infiltrating leukocyte (TIL), leading‐edge region infiltrating leukocyte (LIL), and nontumor region infiltrating leukocyte (NIL). Immune lineages were identified and percentage of which were compared (right). C) tSNE plots of CyTOF data from TIL, LIL, NIL as gated on: PD‐1, ICOS, HLA‐DR, CXCR3, CD161, CD69, CD9. Each dot represents one single cell. Arrows showed distinct expression among three regions. D) The expression of surface molecules showed regional difference in immune lineages.

The immune microenvironment in HCC has been widely studied,^[^ ^12^ ^]^ however, the spatial heterogeneity of immune microenvironment has not been systematically analyzed before. We preformed large‐scale mass cytometry immune profiling of 39 matched tissue specimens from tumor core (referred as T), nontumor region (referred as N), and the leading‐edge area (referred as L, an area with 0.4–0.6 cm distal to the macroscopic malignant‐benign boundary from both directions) of 13 patients using an immune cell‐centric antibody panel consisting of 35 surface markers (Table S1, Supporting Information). The CyTOF data were processed via online analytic platforms, Cytobank and R packages (**Figure** **1**A). High‐dimensional single‐cell proteomics profiles were collected from nearly 20 000 000 leukocytes (average ≈450 000 cells for each sample). The distributions of immune lineages were visualized in tSNE plots (Figure 1B).

It is observed that the number of T cells progressively reduced from N to T regions in HCC while B cells had the opposite trend. The number of CD16^+^NK cell markedly increased in N region and slightly upregulated in L region compared to T region. The number of monocytes was decreased in N region. The deeper analyses of B cells, NK cells, and monocytes did not find region‐specific cell subclusters with statistical significance (Figure S1A–I, Supporting Information). However, it is observed that several T cell subclusters were significantly enriched in L region according to the cell density map (Figure 1B). Diversified expression patterns of surface markers were observed in different immune cell lineages from different regions (Figure 1C,D). These results indicated the immune microenvironment is spatially heterogeneous in HCCs.

### 2.2 T Cell Clusters Showed Regional Diversity and Displayed Unique Features in L Region

**Figure 2:**
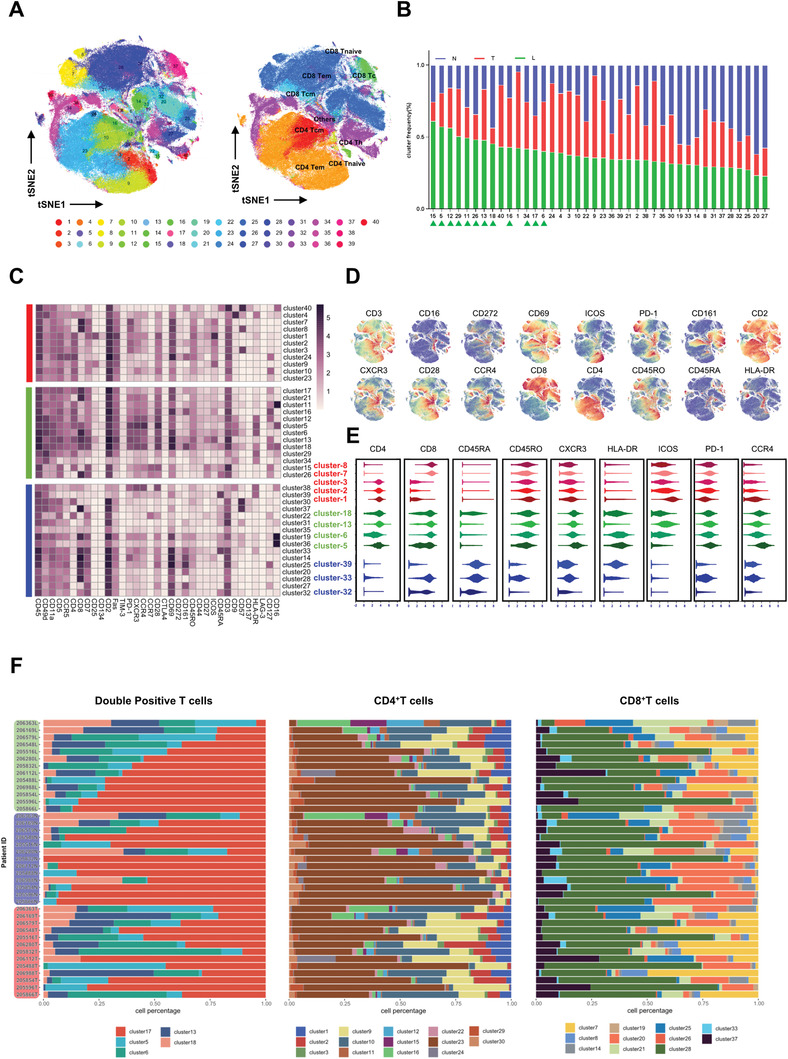
The constitution of T cell clusters showed regional diversity and L region displayed unique immune contexture in terms of T cell. A) tSNE plots showed that 40 clusters were identified in total T cells via FlowSOM clustering method (left). Identified classic T cell subsets were shown on the right. B) Cluster frequency of each of the 40 cluster in T/L/N was shown. The order of the clusters was based on their frequency in L. C) Heatmap showed the mean expression level of all 35 makers in all 40 T clusters. T/L/N enriched clusters were marked on the left. D) tSNE plots of normalized marker expression of T cells from all samples. E) The expression level of several T cell clustering markers and functional markers in T/L/N‐enriched clusters. F) Frequencies of CD8+, CD4+, and DPT clusters for each HCC sample.

To explore the distinct T cell subpopulation composition across the three regions, we visualized and re‐analyzed the T cell subclusters. By applying FlowSOM algorithm, T cells can be partitioned into 40 clusters (including all T cells from the three regions) (**Figure** **2**A and Figure S2A, Supporting Information). Then, we first categorized the 40 clusters into classic T subtypes. It is found that CD4 effector memory T cells (Tem) were gradually increased from N to L (slightly) to T (significantly) regions, and while CD8 Tem showed the opposite trend (Figure S2B,C, Supporting Information). The number of Treg was found decreased from T to L to N as previously reported.^[^ ^7^ ^]^

Among the 40 T cell clusters, 11, 13, and 16 clusters were predominately distributed in T, L, and N regions, respectively (Figure 2B,C and Figure S2D, Supporting Information). The representative expression patterns of surface markers are shown in Figure 2D,E. It is found that several T‐enriched T cell clusters (8, 7, 3, 2, 1) exhibited an exhausted state with a high level of PD‐1 and low expression of HLA‐DR. Many N‐enriched clusters (39, 33, 32) exhibited a rest state with low expression of PD‐1/HLA‐DR and increased CD45RA expression. Interestingly, several L‐enriched clusters (5, 6, 13, 18) showed a “transition” or “bi‐direction” state with an increased expression of both exhaustion (PD‐1, CTLA4) and activation markers (HLA‐DR, ICOS, etc., Figure 2E).

We noticed that DPT cells (including cluster 5, 6, 13, 18) were found enriched in L region of at least 8 out of the 13 HCC patients (>61%) (Figure S2E, Supporting Information). Additionally, we also observed strong phenotype correlation between DPT clusters and clusters 21, 16, 26, 12, 15, and 11 which were all CD28^+^T cells (Figure S2F, Supporting Information).

To further investigate the interpatient heterogeneity of T cells across HCC patients, we examined the frequency of identified T cell clusters within each patient. As shown in Figure 2F, the composition of 5 DPT clusters varied greatly among patients. The similar pattern could also be observed in a few SPT clusters. Taking the patient 206 363 as an example, the lack of cluster 17 of DPT concurred with the decrease of cluster 23 of CD4^+^SPT and cluster 28 of CD8^+^SPT, indicating highly intrapatient heterogeneous immune environment in HCCs.

### 2.3 The Phenotypes and Functions of Tumor‐Associated DPT Cells

**Figure 3:**
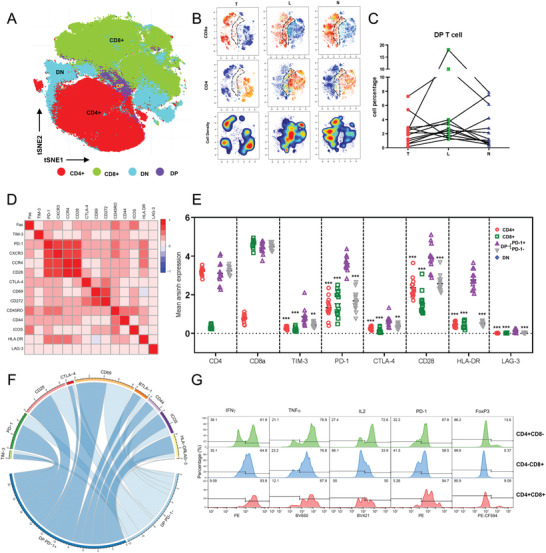
The phenotypes and functions of DPT cells. A) Single positive/double positive/double negative T cells were identified in tSNE plots of total T cells. B) One specific patient (206 363) with significant enrichment of DPT cells in L region. C) Cell percentage of double positive T cells in total T cells from TIL/LIL/NIL of all 13 patients. D) Heatmap showing the coexpression pattern of T cell activation/exhaustion makers in double positive T cells. E) In L region of HCC, the comparison of the expression level of several clustering markers and immune checkpoint molecules was made among single positive/double positive/double negative T cells. Results are shown as mean ± SD, *n* = 13. *P < 0.05, **P < 0.01, ***P < 0.001, based on the Student's *t*‐test. F) The expression intensity of cell activation/exhaustion makers in double positive T cells was shown in circos plots. G) The expression level of IFN*γ*/TNF*α*/IL2/PD‐1/Foxp3 in DPT cells with 4.5h PMA and ionomycin stimulation.

Along the T cell maturation process, classical DPT cells are well‐defined naïve T cells in thymus. However, the molecular features and functions of tumor‐associated DPT cells are poorly studied. To further explore the potential contribution of DPT cells, we separated T cells according to the expression pattern of CD4 and CD8 as CD4 single positive (CD4^+^SPT), CD8 single positive (CD8^+^SPT), double negative (DNT), and double positive (DPT) cells (**Figure** **3**A). As shown in Figure 3B,C, significantly increased DPT cells in L region were found in up to 70% HCC patients (9/13). We further explored the coexpression pattern between T cell activation markers and T cell exhaustion markers in DPT cells. Strong positive coexpressions were observed between PD‐1, CXCR3, CCR4, and CD28 (Figure 3D). Also, it is found that CD45RO, a marker of memory T cell, was positively coexpressed with PD‐1. This coexpression was further confirmed by flow cytometry manual gating plots (Figure S3A,B, Supporting Information). Apart from PD‐1, CD45RO also showed coexpression with TIM‐3 (*r* = 0.23, *P* < 0.0001), CTLA‐4 (*r* = 0.26, *P* < 0.0001), and LAG‐3 (*r* = 0.14, *P* < 0.0001) (Figure S3C, Supporting Information).

PD‐1^+^CD45RO^+^T cells (including cluster 5, 6, 13, 18) accounted for a major subpopulation of DPT cells. And PD‐1^+^CD45RO^+^DPT cells (including cluster 5, 6, 13, 18) were significantly enriched in L region (8/13 patients) (Figure S3D, Supporting Information). It is observed that T cell activation‐related markers including CD28, CD69, CD44, and ICOS were upregulated in PD‐1^+^CD45RO^+^DPT cells, indicating PD‐1^+^CD45RO^+^DPT cells may display an activated phenotype. This PD‐1 expressing and activated phenotype in SPT cells had been reported in lung cancer.^[^ ^13^ ^]^ But the similar scenario in DPT cells was firstly reported here according to our knowledge. Additionally, we found that the phenotype of PD‐1+DPT cells were similar in T/L/N, indicating a similar function of these cells despite their location (Figure S3E, Supporting Information).

Then, we compared the expression levels of immune exhaustion makers between SPT, DPT, and DNT cells. Nearly all immune exhaustion markers including TIM‐3/PD‐1/CTLA‐4/LAG‐3 were significantly upregulated in PD‐1^+^CD45RO^+^DPT cells (Figure 3E,F). However, if we integrated PD‐1^+^CD45RO^+^DPT cells and PD‐1^−^CD45RO^−^DPT cells into one group, this expression difference was largely counteracted (Figure S3F, Supporting Information). By in vitro experiments, we found that upon stimulation with PMA and ionomycin, DPT cells (isolated from L regions) expressed higher level of IFN*γ*, TNF*α* than SPT cells, which clearly verified that DPT cells might stay at an activated stage (Figure 3G). DPT expressed a similar level of IL‐2 as CD8^+^SPT cells, which indicated their ability to activate other T cells. We also found that PD‐1 level was significantly higher in DPT cells, consistent with our defined phenotype of L‐enriched DPT cells. Foxp3 level was relatively higher in CD4^+^SPT and DPT but not CD8^+^Tcells, indicating the existence of Treg‐like DPT cells.

### 2.4 DPT Cells Existed in Various Human Cancers with Similar Phenotype and Served as a Significant Prognostic Factor in HCC

**Figure 4:**
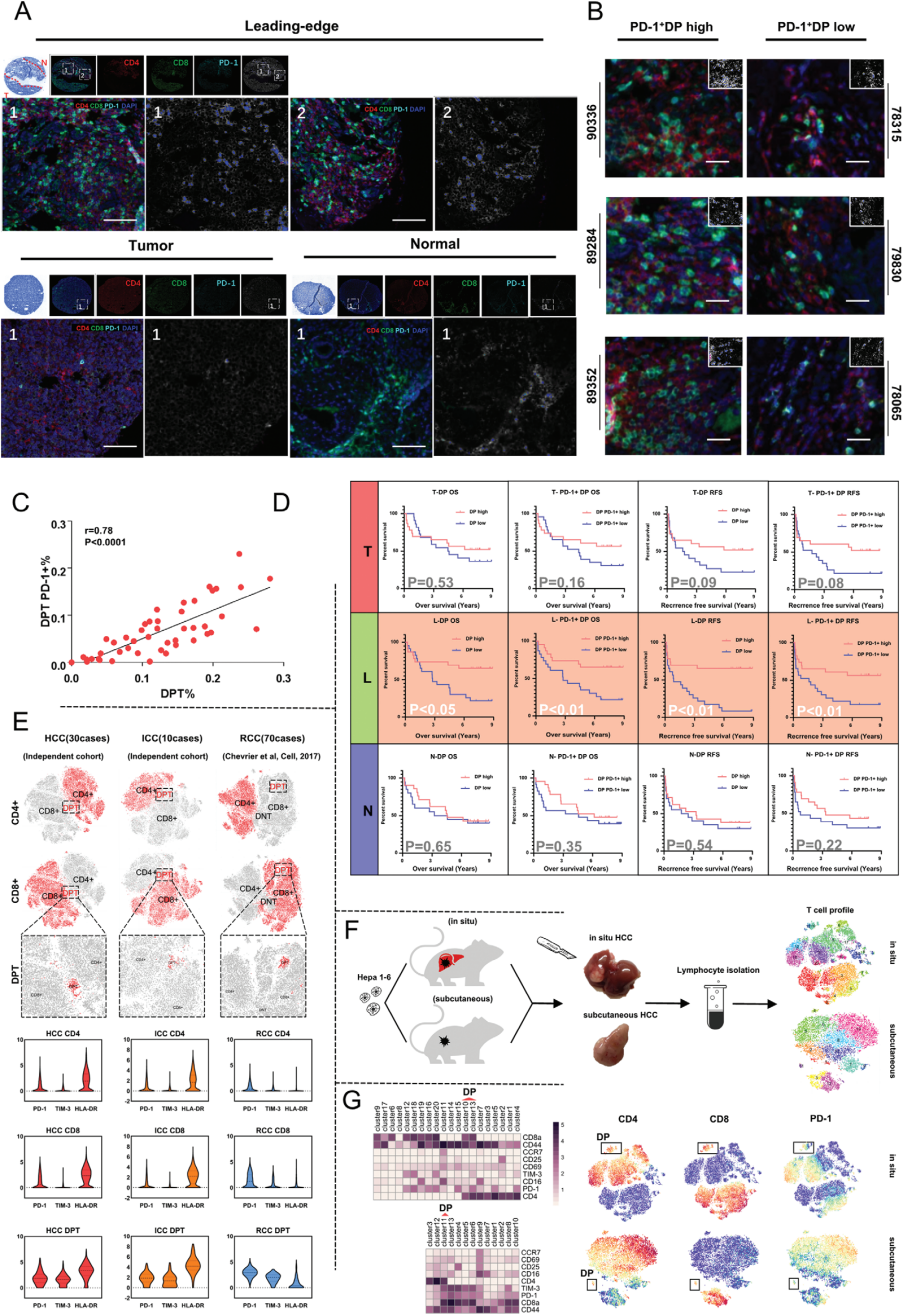
DPT cells existed in various human cancers with similar phenotype and showed prognostic values in HCC. A) Multiplex immunofluorescence staining of CD4^+^ T cells, CD8^+^ T cells, and PD‐1^+^ T cells in HCC tissue microarray. The localization of DP PD‐1^+^ T cells was analyzed with Halo software using Highplex FL module. Scale bar, 100 µm. B) Three exemplified cases of DP PD‐1^+^T high patients and three exemplified cases of DP PD‐1^+^T low patients. Scale bar, 250 µm. The location of triple positive cells is marked in the simulation image at the upper right corner. C) The correlation of cell density between DPT cells and DP PD‐1+T cells. D) Kaplan–Meier analysis of the correlation between DPT/DP PD‐1^+^cell levels and overall survival (OS)/recurrence‐free survival (RFS). E) The existence of DPT cells was confirmed in another three cohorts (HCC, ICC, RCC). 15 000/5000/35 000 cell counts each for analysis. The molecular characterization was shown as below. F) Workflow of in vivo study with in situ HCC model and subcutaneous xenograft model. G) Heatmap showing the expression pattern of mice T cell clusters in different models (left). DPT cells were identified in tSNE plots (right).

To further confirm the spatial distribution of PD‐1^+^DPT cells in vivo, we labeled HCC tissues with CD4/CD8/PD‐1 antibodies simultaneously and examined the sporadic infiltration of T cells in T, L, and N regions (**Figure** **4**A). Consistent with the previous results (Figure 2C), CD8^+^SPT cells mainly resided in N region and CD4^+^SPT cells were mostly spotted in T region. PD‐1^+^DPT cells, which were barely seen in T/N regions, showed extensive existence in L region with strong staining signals of all three antibodies. The numbers of PD‐1^+^DPT (triple positive) cells were counted by HALO software. We found that the densities of PD‐1^+^DPT cells were variable in L regions in different HCC patients (Figure 4B), and were highly correlated with the numbers of DPT cells (Figure 4C).

Previous studies have reported multiple immune populations holding prognostic value in HCC.^[^ ^14^, ^15^, ^16^ ^]^ To examine the potential prognostic value of DPT and PD‐1^+^DPT cells, tissue microarrays consisting of matched T, L, and N specimens from 46 HCC patients were used (Table S2, Supporting Information). Survival analysis showed that more DPT cells and PD‐1^+^DPT cells significantly indicated both better overall survivals and recurrence‐free survivals (Figure 4D). However, this result cannot be observed for T or N region. It is suggested that DPT cells may specifically exert their function in L region. Univariate analysis of survival and recurrence‐related clinicopathological variables showed that DPT cells (HR = 0.35, *P* = 0.016) and PD‐1^+^DPT cell (HR = 0.32, *P* = 0.008) were significantly correlated with overall survival (OS) and recurrence free survival (RFS) (DPT cells: HR = 0.25, *P* = 0.001; PD‐1^+^DPT cell: HR = 0.37, *P* = 0.012). For SPT cells, previous studies reported controversial results of their prognostic values in different types of cancers.^[^ ^17^ ^]^ Our result revealed that CD4^+^SPT cells in T regions were positively associated with OS and RFS, and CD8^+^SPT cells in T regions were unfavorable to prognosis for HCC patients (Figure S4A, Supporting Information).

To further verify the existence and function of DPT cells, we analyzed the T cell profiles of another 30 independent HCC cases and 10 intrahepatic cholangiocarcinoma (ICC) cases collected in our hospital, and 70 renal clear cell carcinoma (RCC) cases reported previously^[^ ^18^ ^]^ (Figure S4B–D, Supporting Information). As reported in the above sections, DPT cells were found in all the three datasets with higher expression of immune checkpoint markers in comparison with SPT cells (Figure 4E). To extend our finding, we verified the existence of DPT cells in two HCC mouse models (in situ/subcutaneous) with Hepa 1–6 cell line (Figure 4F,G). Furthermore, the PD‐1 expression was obviously upregulated in murine DPT. Taken together, these results suggest that DPT cells not only exist in HCCs but also in other tumors.

### 2.5 Characterization of Tumor‐Associated DPT Cells by Single‐Cell RNA‐Seq and TCR‐Seq

**Figure 5:**
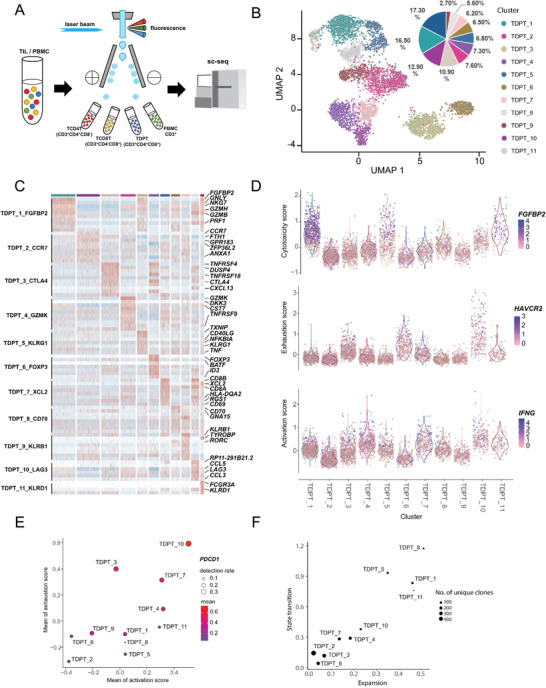
Characterization of tumor‐associated DPT cells by single‐cell RNA‐seq and TCR‐seq. A) Graphic abstract of single‐cell sorting pipeline. B) The tSNE projection of 5018 DPT cells showing the formation of 11 clusters shown in different colors. The functional description of each cluster is determined by the gene expression characteristics. C) Heatmap showing differentially expressed genes of 11 DPT clusters. The top bar indicates cluster ID. Selected differential genes are denoted to the right. D) Cytotoxicity/exhaustion/activation score in all 11 DPT clusters. E) Exhaustion score versus activation score in all 11 DPT clusters. Circle size indicates the detection rate of PDCD1. F) Transition potentials of DPT cell clusters quantified by overall STARTRAC‐tran indices versus expansion potentials of DPT cell clusters quantified by overall STARTRAC‐expa indices. Circle size indicates the number of unique clones.

The detection range of CyTOF antibody panel is limited by the number of available metal isotopes. To further explore the functionalities and evolutionary track of DPT cells, we sorted and performed single‐cell RNA‐seq and TCR‐seq on 5018 tumor‐associated DPT cells (TDPT), 3676 CD4 single positive T cells (TCD4T), 4470 CD8 single positive T cells (TCD8T), and 4652 PBMC CD3^+^T cells (low‐quality cells were filtered before analysis) (**Figure** **5**A and Figure S5A, Supporting Information). The scRNA‐seq analysis identified 11 cell subpopulations of DPT cells (Figure 5B). TDPT_1–4 accounted for over 50% of all DPT cells in total, while the remaining seven DPT clusters each accounted for less than 8% (Figure 5B). TDPT_1_FGFBP2 specifically expressed “cytotoxic” marker gene such as *FGFBP2*, *GNLY*, and *GZMH*. TDPT_2_CCR7 was characterized by its naïve nature with increased expression of *CCR7*. TDPT_3_CTLA4 uniquely expressed high level of *CTLA4* along with *TNFRSF4/18*. TDPT_4_GZMK expressed high level of *GZMK* and *TNFRSF9*. TDPT_5_KLRG1 had an increased expression of *KLRG1* along with cytotoxic genes (*TNF/NFKBIA*). TDPT_6_FOXP3 exhibited Treg properties by expressing high level of *FOXP3* and *CTLA4*. TDPT_7_XCL2 showed T memory properties by expressing high level of *XCL2/CD69*. TDPT_8_CD70 expressed high level of *CD70*, an immune activation marker. TDPT_9_KLRB1 was transcriptomically similar with *MAIT*. TDPT_10_LAG3 expressed high level of *LAG3* and *CTLA4* along with *GZMH*, *GZMB*, *CCL3*, and *CCL5*. TDPT_11_KLRD1 was transcriptomically similar with NKT (Figure 5C).

To examine the phenotypes of different DPT clusters, we scored cells by cytotoxic, activation,^[^ ^19^ ^]^ and exhaustion signatures^[^ ^20^ ^]^ based on the top 20 genes correlated with FGFBP2, IFNG, and HAVCR2 expression, respectively. For CD8‐like clusters, we found that DPT cells with a high cytotoxic signature (TDPT_1_FGFBP2, TDPT_5_KLRG1, TDPT_11_KLRD1) usually expressed a low level of exhaustion signature and a high level of activation signature, except for TDPT_10_LAG3 which had a high level of all three signatures. TDPT_3_CTLA4 and TDPT_6_FOXP3 both had a high exhaustion signature accompanied by low cytotoxic and activation signatures (Figure 5D). We then examined the link between the cytotoxic and exhaustion signature in all DPT clusters and observed that most DPT clusters exhibited either high cytotoxic signature or exhaustion signature except for TDPT_2_CCR7 and TDPT_10_LAG3 (Figure 5E). TDPT_2_CCR7 had both low cytotoxic and exhaustion signature, consistent with its undifferentiated state, while TDPT_10_LAG3 showed the highest expression of both cytotoxic and exhaustion signatures. Furthermore, TDPT_10_LAG3 expressed the highest level of PDCD1 (Figure 5E). Collectively, TDPT_10_LAG3 was phenotypically consistent with the PD‐1^+^DPT cells defined by the previous CyTOF analysis.

By analyzing TCR clonotypes across the DPT clusters (except TDPT_9_KLRB1 which has distinct TCRs), we found that TDPT_2_CCR7, TDPT_3_CTLA4, and TDPT_6_FOXP3 harbor largest number of diverse TCR clonotypes with the least expansion tendency^[^ ^21^ ^]^ (Figure 5F). Oppositely, TDPT_1_FGFBP2, TDPT_5_KLRG1, TDPT_8_CD70, and TDPT_11_KLRD1 harbor the fewest TCR clonotypes but with the highest expansion tendency. It was observed that the cytotoxicity and activation of DPT cells were positively correlated with TCR expansion, while undifferentiation and exhaustion showed negative correlation. As to state transition,^[^ ^21^ ^]^ cytotoxic and activated DPTs were prone to change their transcriptional state while naïve and exhausted DPTs showed less such tendency. TDPT_10_LAG3 with both high level of activation and exhaustion score showed moderate expansion and transition tendency (Figure 5F).

### 2.6 Tumor‐Associated DPT Cells Originated from Infiltrating SPT Cells

As classical T cell maturation process, CD4/CD8 double positive T cells were well‐described naïve T cells within thymus.^[^ ^11^ ^]^ Although DPT cells in blood and peripheral lymphoid tissues have been widely studied, their molecular features and cellular functions in cancers are unclear.^[^ ^22^ ^]^ Based on CyTOF data, as shown in Figure S6A (Supporting Information), the 3D diffusion plots had two major branches (T cell clusters were labeled according to the above results by FlowSOM analysis). The expression intensity of single surface markers was also drawn (Figure S6B, Supporting Information). We observed that each of the two branches exclusively expressed CD4 or CD8 and the merging point of these two branches was the location of DPT cells (cluster 5, 6, 13, 17, 18). The coexpression of CD45RO and PD‐1 was found highest in DPT cells and faded along the two branches. Other immune exhaustion markers (TIM3 and CTLA‐4) also showed slightly increased expression in DPT cells except for LAG‐3 because of its relatively low expression level (Figure S6B, Supporting Information). Interestingly, the expression of CD45RA, the surface marker for naïve T cells was markedly increased at the ending points of the two branches. As shown in Figure S6C (Supporting Information), CD4 or CD8 branch expanded toward opposite directions along diffusion map component 2 (DC2). The expression of CD45RO and PD‐1 showed great consistency along DC2 axis and the peak of both appeared at CD4/CD8 double positive region (Figure S6C, Supporting Information). It can be inferred that naïve T cells located at the two terminals differentiate into the middle T cells (Figure S6D, Supporting Information). Additionally, we found that PD‐1^+^CD8^+^SPT cells (cluster 8) were very close to DPT (cluster 5, 6, 13, 18), implying a close evolutionary relationship between them.

**Figure 6:**
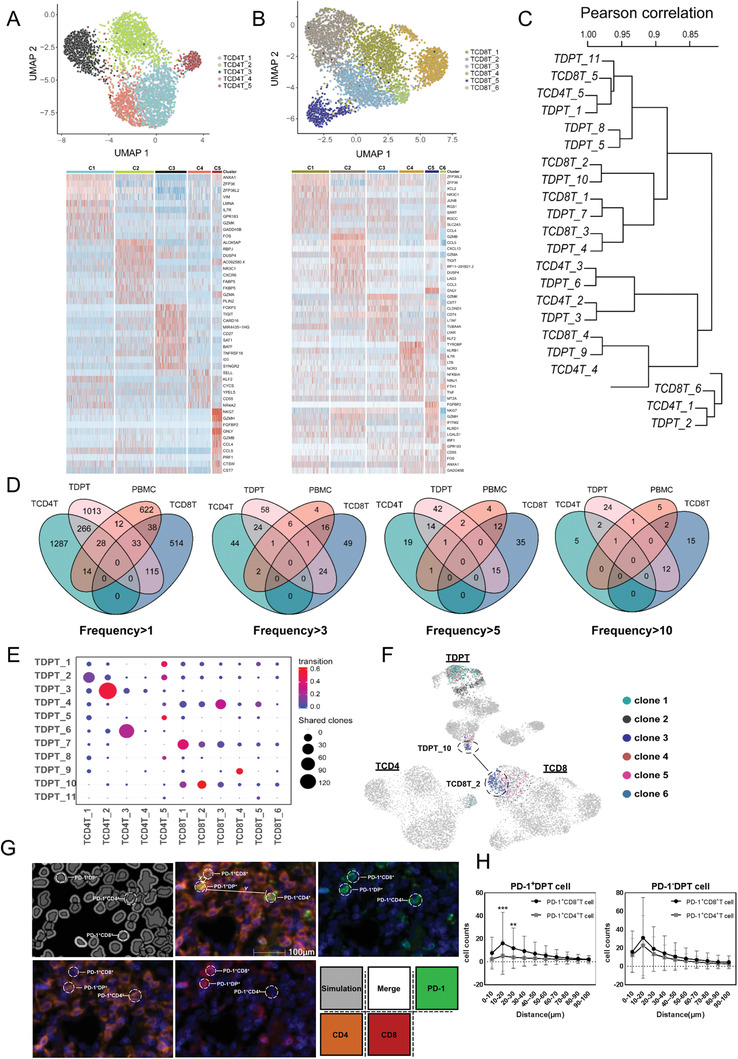
Tumor‐associated DPT cells originated from infiltrating SPT cells. A,B) The tSNE projection of 3676 CD4^+^T/4470 CD8^+^T cells (above). Heatmap showing differentially expressed genes of 5 CD4^+^/6 CD8^+^ clusters. The top bar indicates cluster ID (below). C) The transcriptomic similarity was compared between DPT and SPT clusters. D) Venn diagram shows the overlap of TCR clonotypes among TDPT, TSPT, and PBMC CD3^+^ T cells. E) The number of shared TCR clones between DPT clusters and SPT clusters. The transition potentials were color‐coded. F) Top 6 TCR clones with the highest frequency were highlighted on UMAP of DPT and SPT cells. G) Examples of distances between a DPT PD‐1+cell (center) and other neighboring CD4/CD8 PD‐1+T cells with 15 µm (*x*), 45 µm (*y*). H) The cell counts of CD4+/CD8+ PD‐1+T cells within different distances of DP PD‐1+T cells/DP PD‐1‐T cells. Results are shown as mean ± SD. *P < 0.05, **P < 0.01, ***P < 0.001, based on the Student's *t*‐test.

To further confirm above observations, we sorted and performed scRNA‐seq analysis on single positive T (SPT) cells, including 3676 tumor‐associated CD4^+^T cells and 4470 CD8^+^T cells. CD4^+^T cells and CD8^+^T cells were divided into five and six clusters, respectively (**Figure** **6**A,B). We analyzed the transcriptomic similarity (Pearson correlation of signature gene expressions) between clusters of DPT cells and SPT cells (Figure 6C). TDPT_1/5/8 were similar with TCD4T_5 and TCD8T_5 which expressed high level of *NKG7/GZMH/FGFBP2*. TDPT_10 was similar with TCD8T_2 which expressed high level of *TIGIT/LAG3/GZMA/GZMB*. TDPT_2 shared phenotypical similarities with TCD4T_1 and TCD8T_6 which exhibited naïve property.

TCR analysis revealed that TDPT cells shared more TCR clones with TCD4 and TCD8 than PBMC, indicating tumor‐associated DPT cells were most likely transformed from intratumoral SPT cells (Figure 6D). Interestingly, when we checked the shared clones with high frequency, we found that DPT cells mainly shared high‐frequency TCR clones with CD8^+^T cells. To further identify the position of DPT cells in T cell evolutionary track, we checked the shared clones between DPT and SPT clusters (Figure 6E). The most shared TCR clones existed between TDPT_3/TCD4T_2 and TDPT_6/TCD4T_3. Notably, they also shared similar transcriptome pattern. TDPT_10 mainly shared its TCR clones with TCD8T_2 which expressed high level of both exhaustion and cytotoxicity genes, indicating TDPT_10 was a functional T cell cluster originated from SPT cells. The transition score further indicated that TDPT_3 and TDPT_10 were robustly changing their transcriptomic patterns. We projected the top six TCR clones with the highest frequency on UMAP of DPT/SPT (Figure 6F). Interestingly, most of these clones existed in DPT cells and specifically in cytotoxic and activated DPT clusters (C1, C5, C8) and TDPT_10, indicating a TCR expansion state.

By applying multiplex immunofluorescence tissue staining, we also observed that PD‐1^+^CD8^+^SPT cells are spatially closer to PD‐1^+^DPT cells than PD‐1^+^CD4^+^SPT cells, while no such difference was found in PD‐1^−^DPT cells (Figure 6G,H). Taken together, our data here delineated the phenotypic developmental path of T cell and suggest that PD‐1^+^DPT cells derived from intratumoral CD8^+^ T cells, rather than PD‐1^+^CD4^+^ or naïve T cells in thymus.

## 3 Discussion

Immune microenvironments show high heterogeneities across different cancers and high diversity among different individuals.^[^ ^23^ ^]^ The exploration of the density, location, and function of different immune components in human tumors has led to the identification of the key protumor and antitumor immune signatures. Histopathological analyses of various tumors have confirmed that the distribution of infiltrated immune cells shows great differences within the tumor and its surrounding nontumor regions.^[^ ^24^ ^]^ Crosstalks between tumor cells and adjacent stroma cells have been identified as an important driver for tumor progression.^[^ ^25^ ^]^ Stroma‐induced inflammation and angiogenesis have been proved to have a tumor‐promoting role.^[^ ^4^ ^]^ In a work by Chew et al.,^[^ ^7^ ^]^ they revealed the existence of an immunosuppressive gradient across the tumor microenvironment, nontumor microenvironment, and peripheral blood in HCC. Whereas, the characteristic immune microenvironment of the leading‐edge region, a transition region from the tumor core to the adjacent nontumor tissue, has not been well studied. Compared with the work by Chew et al.,^[^ ^7^ ^]^ we intended to focus more on the intratumoral immune heterogeneity. To this end, we acquired the surface markers profile of 17 432 600 immune cells from 39 paired T/L/N samples from 13 HCC patients and discovered distinct L‐region specific immune cell signatures. In terms of T cells, CD4 and CD8 double positive T (DPT) cells, expressing both exhaustion and activation markers, are significantly enriched in L regions in several patients. Previously, double positive T cells were described as a premature developmental stage within the thymus.^[^ ^11^ ^]^ It was generally believed that once differentiated, the CD4^+^ lineage and CD8^+^ lineage are determined for a single cell. Although mature DPT cells have been reported in certain disease conditions, such as melanoma^[^ ^26^ ^]^ and lymphoma,^[^ ^27^ ^]^ little is known about its origin and potential biological functions. In our study, we firstly reported that most of L‐enriched DPT clusters are phenotypically active and memory‐like with enhanced expression of PD‐1/CD45RO expression. DPT cells can be further divided into PD‐1^+^CD45RO^+^ DPT and PD‐1^−^CD45RO^−^ DPT subtypes. It has been reported that single positive T effector memory cells coexpressing PD‐1 with multiple immune checkpoint molecules actually present a hyperactivated phenotype.^[^ ^13^ ^]^ Here, PD‐1^+^CD45RO^+^ DPT cells also express T cell activation markers which indicate that they are proliferating and functional T cells. In vitro study, after stimulation, DPT cells exhibited a strong immune response as SPT cells, confirming their active antitumor state. Previous studies have reported various immune populations exhibiting prognosis impact in HCC, including Treg,^[^ ^15^ ^]^ tumor‐associated macrophage,^[^ ^16^ ^]^ and resident natural killer cells.^[^ ^14^ ^]^ We found that L region‐enriched DPT cells was positively associated with a better outcome in HCC patients. Collectively, our data here implied that the potential antitumor activity of DPT cells for HCC patients.

To further investigate the diversity in L‐enriched DPT cells, single cell sequencing was applied and discovered 11 DPT clusters. Based on the unique gene signatures, we identified cytotoxic DPT cells (TDPT_1 and TDPT_5) with strong expression of *FGFBP2/GNLY/GZMH/GZMB/KLRG1/TNF*, memory DPT cells (TDPT_4 and TDPT_7) with strong expression of *GZMK/XCL2/CD69*, DPT reg cells with high expression of *FOXP3*, and activated DPT cells with high expression of *CD70*. DPT‐NK and DPT‐MAIT expressed high level of *KLRD1* and *KLRB1*, respectively. The identity of DPT‐MAIT was further verified by TCR‐seq data. Interestingly, we also discovered DPT clusters (TDPT_3 and TDPT_10) expressing high level of T cell exhaustion genes. Further investigation revealed that TDPT_10 exhibited high levels of cytotoxicity and activation while TDPT_3 showed low levels of these characteristics. The phenotype of TDPT_10 showed high resemblances to CyTOF‐defined PD‐1^+^DPT cells which proved our hypothesis that PD‐1^+^DPT cells were functional activated cells rather than premature T cells.

To identify the position of L‐enriched DPT cells in the developmental trajectory of T cells, we applied diffusion map to demonstrate the phenotypic evolution of T cells in HCC. It turned out that PD‐1^+^CD45RO^+^DPT cells were not the early developmental stage of T cells, instead, these cells were functional and well‐differentiated T cells. TCR‐seq data suggested that PD‐1^+^DPT cells shared most TCR clonotypes with PD‐1^+^CD8^+^T cells, indicating that PD‐1^+^DPT cells shared the same ancestry with PD‐1^+^CD8^+^SPT cells and originated from intratumoral CD8^+^SPT cells which is in line with previous studies that CD8^+^T cells can be transformed into mature, functional DPT cells under certain conditions.^[^ ^28^ ^]^ Further studies with parallel analysis of shared TCR clonotypes between TDPT and PBMC CD3^+^T cells confirmed the local origin of most TDPT cells.

In conclusion, we defined a unique DPT‐enriched immune microenvironment in the L area of HCC. With a combined technique of CyTOF and single‐cell sequencing, diversified DPT clusters were identified with different functions. PD‐1^+^DPT cells exhibit an activated state with strong expression of both exhaustion and activation markers and hold potential prognostic value in HCC patients. TCR analysis revealed that the origin of PD‐1^+^DPT cells shared the same ancestry with PD‐1^+^CD8^+^SPT cells and were most likely transformed from intratumoral SPT cells. Our study delineated the complex functionalities in HCC DPT cells and provides a new evolutionary finding for DPT cells.

## 4 Experimental Section

##### Mass Cytometry by Time‐of‐Flight and Data Processing

Thirteen groups of TILs, LILs, and NILs were obtained from HCC patients as described before and processed for CyTOF analysis. A panel of 35 antibodies that encompassed a broad range of immune subsets was used together (Table S1, Supporting Information). The preconjugated antibodies were purchased directly from the supplier. Leukocytes were washed and stained for viability with 10 × 10^−3^ m cisplatin for 2 min to identify live/dead cells and incubated with metal‐conjugated surface‐membrane antibodies for 30 min at 37 °C. After that, cells were fixed with fix perm buffer. Finally, a cell intercalation (mixture of fix perm buffer and iridium) was added for cellular fixation and visualization and this procedure lasted for overnight before analysis on a Helios mass cytometer (Fludigm, USA). EQ Four Element Calibration Beads were used according to manufacturer's instructions to normalize the signal. 250 000–500 000 cell events were collected for each sample. Files (.fcs) were uploaded into Cytobank, populations of interest were manually gated, and events of interest were exported as .fcs. files. For further analysis, a random sampling of 6000 cells from each fcs file was performed using a CyTOFkit program on R. Visualizations based on tSNE and clustering based on the FlowSOM/Renograph algorithms were then performed on these cells.

##### Single‐Cell RNA‐Seq and Data Processing

Immune cells were sorted via flow cytometry before submitted to single‐cell sequencing platform. The chromium single cell expression solution (10× genomics) was used to generate single cell transcriptomes. Single cell suspensions were loaded onto the Chromium Controller (using the protocol of Chromium Single Cell 5′ Library system). The libraries were sequenced by HiSeq4000 (Illumina) (150 bp paired‐end sequencing).

The CellRanger (3.1.0) software was used to map the raw sequences to the human GRCh38 genome, remove empty beads, and generate the raw UMI counts matrix. The R (3.6.0) package “Seurat”^[^ ^29^ ^]^ (3.1.1) was then used to perform the following analyses. First, low‐quality cells were removed. Four quality control indices were adopted: the number of UMIs, the number of detected genes, the proportion of UMIs derived from mitochondrial genes, and the proportion of UMIs derived from dissociation or sorting associated genes. For each index, the boxplot of its distribution over cells was generated, and those cells identified as upper outliers were regarded as low‐quality cells. Cells with less than 200 detected genes were also removed. Initial clustering identified some non‐T cells, which were not used for following analysis. Next, for each cell, the TPM‐like value for each gene was obtained by dividing the sum of UMI counts in the cell and multiplying 10 000. The TPM‐like values were further logarithm transformed after adding a pseudo‐count 1. Highly variable genes (2000) were detected by the function “FindVariableFeatures.” Previous studies showed clustering results may be significantly influenced by the ribosome genes.^[^ ^30^ ^]^ Ribosome genes and heat shock response genes (from GO term “GO_CELLULAR_RESPONSE_TO_HEAT”) were excluded from the highly variable gene list. The number of UMIs and the percent of mitochondrial genes were regarded as the confounding factors and regressed out. Then the expression matrix was scaled and principal component analysis was performed on the submatrix consisting of the highly variable genes. The top 20 principal components were used for clustering analysis and visualization. The “FindNeighbors” and “FindClusters” functions (resolution set as 0.8) were used to perform clustering. The “RunUMAP” function was used to generate the 2D visualizations. The “FindAllMarkers” function was used to detect differentially expressed genes between clusters.

##### TCR Profiling

TCR clonotype for each cell was extracted by cellranger. Clonotypes are defined by the CDR3 (the complementarity determining region 3) sequences. Three indices were then adapted from STARTRAC^[^ ^16^ ^]^ to analyze the clonotypes of T cells clusters: 1) STARTRAC‐expa (measure the degree of clonal expansion), 2) STARTRAC‐tran (measure the degree of state transition), and 3) pSTARTRAC‐tran (pairwise state transition of two clusters). All these indices are basically defined based on the Shannon entropy, and higher values indicate higher clonal expansion or state transition.

##### Patients and Sample Processing

HCC tissues with a size of 6 cm (length) × 4 cm (width) × 3 cm (thickness) were obtained from a total of 13 HCC patients (patients’ information was listed in Table S3, Supporting Information) from the Eastern Hepatobiliary Surgery Hospital, who underwent curative surgical resection for HCC. Informed consent was obtained from all patients and the procedure of human specimen collection was approved by the Ethics Committee of Eastern Hepatobiliary Surgery Hospital (Shanghai, China). To accurately obtain the leading‐edge region, an area was isolated, 0.4–0.6 cm distal to the macroscopic malignant‐benign boundary from both directions and defined it as L region. The remaining two tissue parts were defined as T and N based on their histological features. Single cell suspension was isolated by enzymatic digestion. All samples were anonymously coded in accordance with local ethical guidelines.

##### Isolation of Leukocytes from Tissues–Digestive Enzyme

The digestive enzyme dissolved in RPMI with 10% serum was composed of collagenase IV, deoxyribonuclease type I, and hyaluronidase type V.

##### Isolation of Leukocytes from Tissues–Tissue Digestion

The tissues washed by Hank's balanced salt solution (HBSS) were minced and digested by digestive enzyme, shaken for 60 min at 37 °C, and then filtered through the 300‐mesh filter screen. The filtered mixture was then collected in 50 mL centrifuge tubes.

##### Isolation of Leukocytes from Tissues–Density Gradient Centrifugation

Filtered mixture collected above were centrifuged with 450 *g* for 8 min and then the precipitates were centrifuged with 50 *g* for 1 min after being resuspended by HBSS. Carefully superimpose the clear supernatant on the surface of lymphoprep liquid and then centrifuge with 450 *g* for 25 min. Leukocytes were concentrated in the middle layer of the mixed liquid after being centrifuged.

##### Multiplex Immunofluorescence Tissue Staining

Two tissue microarrays containing T/L/N samples of 52 patients were stained with Opal Multiplex Immunohistochemistry Detection Kit (Perkin‐Elmer) and images were acquired using a Vectra 3.0 Pathology Imaging System Microscope (Perkin‐Elmer). Slides were deparaffinized and rehydrated and antigen retrieved using Trilogy buffer (CellMarque) by autoclaving for 15 min. Slides were treated with 3% H_2_O_2_ for 15 min, washed, and blocked using 4% BSA/PBS/0.1% Triton X‐100 (all from Sigma). Antibodies used were: anti‐CD8, anti‐CD4, and anti‐PD‐1. Detection dye for each antibody was: Opal570 dye (CD8), Opal520 dye (CD4), and Opal620 dye (PD‐1). DAPI was used as a nuclear counterstain. The digital images were analyzed with Halo Image Analysis software (indica labs) using Highplex FL module which allows for the simultaneous analysis of up to eight immunofluorescence‐labeled markers in any cellular compartment—nucleus, cytoplasm, and/or membrane. Cells negative for all markers are black, cells positive for individual makers are colored according to that marker color, and cells positive for three markers were calculated and marked in blue in the simulation image. Antibodies used in this experiment and the corresponding dilution ratio were listed in Table S4 (Supporting Information).

##### Real‐Time PCR

Total RNA was isolated from cells using the RNeasy Mciro kit (Qiagen) according to the manufacturer's protocols. Real‐time PCR analyses were performed using an ABI 7300 Fast Real‐Time PCR System (Applied Biosystems, Foster City, CA) and SYBR Green PCR kit (Applied TaKaRa, Otsu, Shiga, Japan). The ΔCt method was used with actin mRNA as an endogenous control for normalization of the results. Primers used in the study were listed in Table S5 (Supporting Information).

##### Animal Models

All animal experiments were performed in accordance with the National Institutes of Health guidelines and approved by the animal care and use committee of the Second Military Medical University. 106 Hepa 1–6 cells were injected subcutaneously or directly to the left lobe of livers of C57BL/6J mice. Mice were sacrificed for tumor collection after three weeks. Harvested tumors were then extracted for immune cells and stained with metal‐labeled antibodies (Table S1, Supporting Information).

##### Flow Cytometry Analysis

Mononuclear cells were isolated from human HCC the borderline region (L) with Lymphoprep following manufacturer's instructions. Cells were collected and washed. After stimulating with Phorbol 12‐myristate 13‐acetate, Ionomycin, Brefeldin A for 4.5 h, the samples were stained with surface markers mouse anti‐human APC‐Cy7 conjugated CD45 (clone 2D1), Alexa Fluor 700 conjugated CD3 (clone UCHT1), BB700 conjugated CD4 (clone SK3), BB515 conjugated CD8 (clon3 RPA‐T8), and PE conjugated PD‐1 (clone EH12.1) (BD Biosciences). Cells were then incubated with Transcription Factor Fixation/Permeabilization working solution (Biogems) for 30 min at 4 °C, washed with Permeabilization working solution (Biogems) twice, and stained with mouse antihuman BV421 conjugated IL2 (clone 5344.111), BV650 conjugated TNF*α* (clone MAb11), PE conjugated IFN*γ* (clone 4S.B3), and PE‐CF594 conjugated FoxP3 (clone 259D/C7) (BD Biosciences). The samples were either stained with anti‐CD45 + CD3 + CD4 + CD8 + IL2 + TNF*α* + IFN*γ*, with anti‐CD45 + CD3 + CD4 + CD8 + IL2 + TNF*α*+ PD‐1, or with anti‐CD45 + CD3 + CD4 + CD8 + IL2 + TNF + FoxP3. After washing with Permeabilization working solution twice, the samples were subjected to flow cytometry analysis (BD LSRFortessa). The data were further analyzed with FlowJo vX.07 software (Tree Star). Antibodies used in this experiment were listed in Table S6 (Supporting Information).

##### Data and Software Availability

The accession number for the CyTOF data reported in this paper and related data had been uploaded onto Mendeley Database: https://doi.org/10.17632/jxsz3hdsyg.2. The sequencing data presented in this paper are available for download on GSA data repository accession numbers: CRA001276 (Temporary link for review: http://bigd.big.ac.cn/gsa/s/0Sla2C2N). Software used in this study was listed in Table S1 (Supporting Information).

## Conflict of interest

The authors declare no conflict of interest.

## Author Contributions

B.Z., D.F.W., X.Y.Q., and G.J.L. contributed equally to this work. B.Z., D.F.W., X.Y.Q., and G.J.L. developed the concept and discussed experiments; B.Z. and D.F.W. designed, performed, and analyzed experiments, and wrote the manuscript; T.W. and S.Y. contributed to the bioinformatic analyses; Y.J.Z., X.W., R.W., Y.G., C.J.S., and Z.X.L. processed patients’ samples and provided technical assistance; S.W., Z.Q.G., and S.Y.S. provided technical assistance; B.Z. and S.S.J. performed statistical analyses of survival in patients; J.G., H.Y.W. and L.C. supervised the progress of the study and edited the manuscript.

## Supporting information

Supporting Information
