# Supplementary material for: Trajectory and Functional Analysis of PD‐1high CD4+CD8+ T Cells in Hepatocellular Carcinoma by Single‐Cell Cytometry and Transcriptome Sequencing
Source: Adv Sci (Weinh). 2020 May 18;7(13):2000224. doi: 10.1002/advs.202000224 (PMC7341083; doi:10.1002/advs.202000224)
Supplement: Supplementary file 1 — Supporting Information [file ADVS-7-2000224-s001.pdf]

Supporting information:

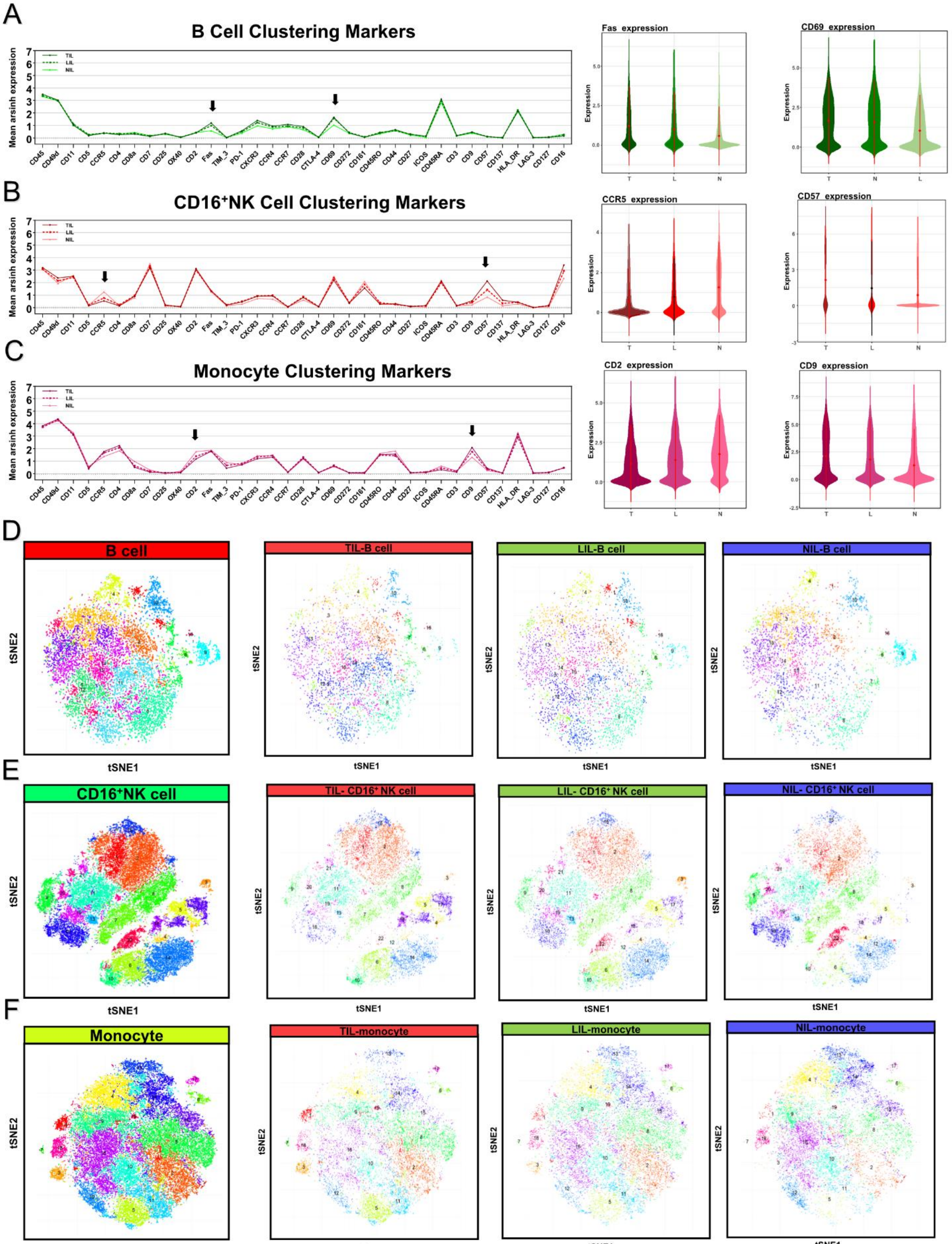

A
B Cell Clustering Markers
Fas expression
CD69 expression
B
CD16+NK Cell Clustering Markers
CCR5 expression
CD57 expression
C
Monocyte Clustering Markers
CD2 expression
CD9 expression
Mean arsinh expression
Expression
TIL
LIL
NIL
D
B cell
TIL-B cell
LIL-B cell
NIL-B cell
E
CD16+NK cell
TIL- CD16+ NK cell
LIL- CD16+ NK cell
NIL- CD16+ NK cell
F
Monocyte
TIL-monocyte
LIL-monocyte
NIL-monocyte
tSNE1
tSNE2

G

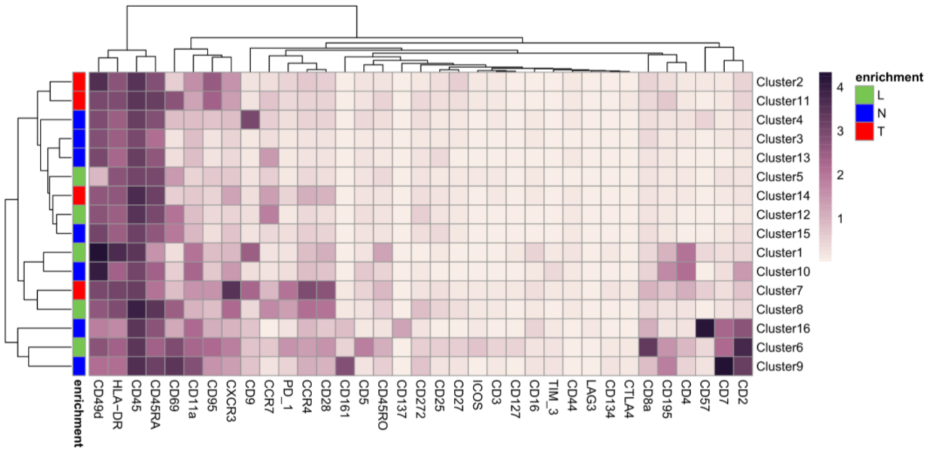
enrichment
L
N
T
4
3
2
1
Cluster2
Cluster11
Cluster4
Cluster3
Cluster13
Cluster5
Cluster14
Cluster12
Cluster15
Cluster1
Cluster10
Cluster7
Cluster8
Cluster16
Cluster6
Cluster9
enrichment
CD49d
HLA−DR
CD45
CD45RA
CD69
CD11a
CD95
CXCR3
CD9
CCR7
PD_1
CCR4
CD28
CD161
CD5
CD45RO
CD137
CD272
CD25
CD27
ICOS
CD3
CD127
CD16
TIM_3
CD44
LAG3
CD134
CTLA4
CD8a
CD195
CD4
CD57
CD7
CD2

H

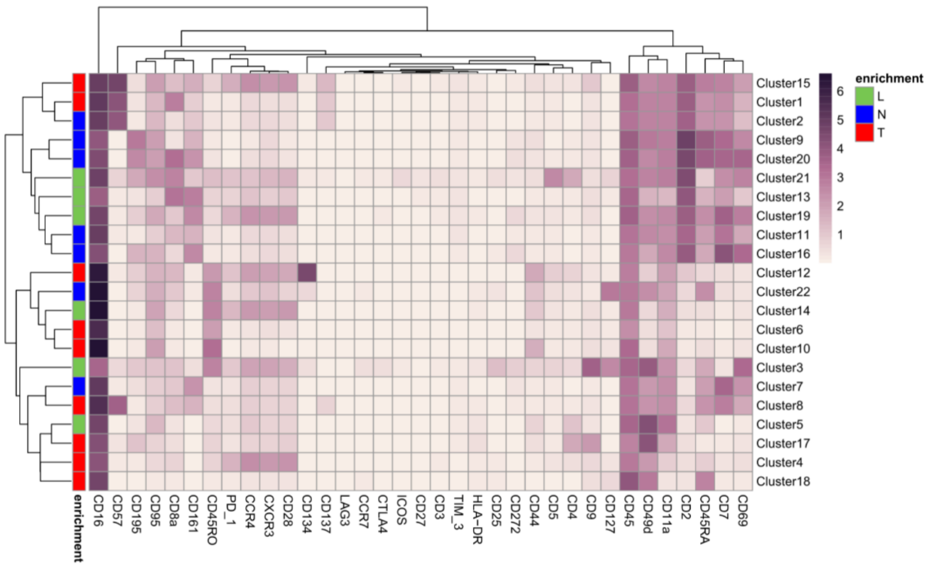
enrichment
L
N
T
6
5
4
3
2
1
Cluster15
Cluster1
Cluster2
Cluster9
Cluster20
Cluster21
Cluster13
Cluster19
Cluster11
Cluster16
Cluster12
Cluster22
Cluster14
Cluster6
Cluster10
Cluster3
Cluster7
Cluster8
Cluster5
Cluster17
Cluster4
Cluster18
enrichment
CD16
CD57
CD195
CD95
CD8a
CD161
CD45RO
PD_1
CCR4
CXCR3
CD28
CD134
CD137
LAG3
CCR7
CTLA4
ICOS
CD27
CD3
TIM_3
HLA−DR
CD25
CD272
CD44
CD5
CD4
CD9
CD127
CD45
CD49d
CD11a
CD2
CD45RA
CD7
CD69

I

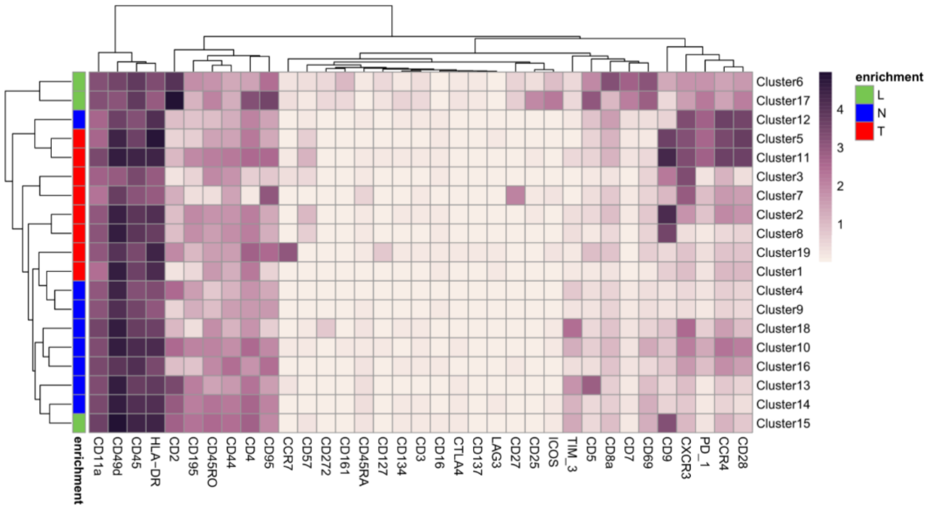
enrichment
L
N
T
4
3
2
1
Cluster6
Cluster17
Cluster12
Cluster5
Cluster11
Cluster3
Cluster7
Cluster2
Cluster8
Cluster19
Cluster1
Cluster4
Cluster9
Cluster18
Cluster10
Cluster16
Cluster13
Cluster14
Cluster15
enrichment
CD11a
CD49d
CD45
HLA−DR
CD2
CD195
CD45RO
CD44
CD4
CD95
CCR7
CD57
CD272
CD161
CD45RA
CD127
CD134
CD3
CD16
CTLA4
CD137
LAG3
CD27
CD25
ICOS
TIM_3
CD5
CD8a
CD7
CD69
CD9
CXCR3
PD_1
CCR4
CD28

Figure S1 A. Average mean expression levels of each of the 3 different regions in HCC were shown for the 35 clustering proteins in B cells (left). Certain differentially expressed markers (Fas, CD69) were shown in violin plots (right). B. Average mean expression levels of each of the 3 different regions in HCC were shown for the 35 clustering proteins in NK cells (left). Certain differentially expressed markers (CCR5, CD57) were shown in violin plots (right). C. Average mean expression levels of each of the 3 different regions in HCC were shown for the 35 clustering proteins in Monocyte (left). Certain differentially expressed markers (CD2, CD9) were shown in violin plots (right). D. tSNE plots of B cells. B cells from three regions were shown in merge (left) and separately (right). E. tSNE plots of CD16+NK cell. CD16+NKs from three regions were shown in merge (left) and separately (right). F. tSNE plots of monocytes. Monocytes from three regions were shown in merge (left) and separately (right). G. Heatmap showed the mean expression level of all 35 makers in all B clusters. Cluster analysis was performed on both columns and rows. T/L/N enriched clusters were marked on the right. H. Heatmap showed the mean expression level of all 35 makers in all CD16+NK clusters. Cluster analysis was performed on both columns and rows. T/L/N enriched clusters were marked on the right. I. Heatmap showed the mean expression level of all 35 makers in all monocyte clusters. Cluster analysis was performed on both columns and rows. T/L/N enriched clusters were marked on the right.

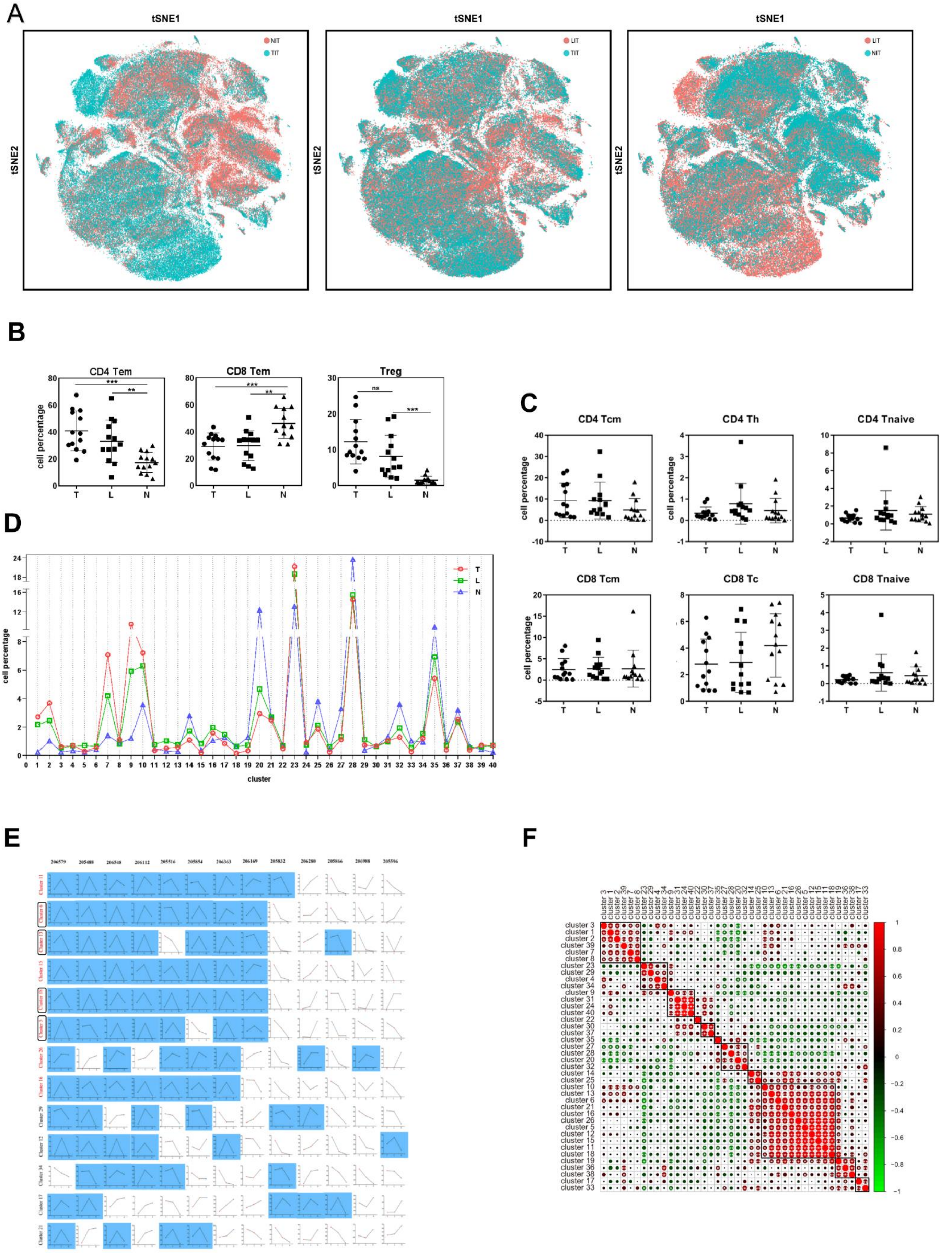
A
tSNE1
tSNE2
NIT
TIT
LIT
B
CD4 Tem
CD8 Tem
Treg
cell percentage
T
L
N
ns
C
CD4 Tcm
CD4 Th
CD4 Tnaive
CD8 Tcm
CD8 Tc
CD8 Tnaive
D
cell percentage
cluster
E
206579
205488
206548
206112
205516
205854
206363
206169
205832
206280
205866
206988
205596
F
cluster 3
cluster 1
cluster 2
cluster 39
cluster 7
cluster 8
cluster 23
cluster 29
cluster 4
cluster 34
cluster 9
cluster 31
cluster 24
cluster 40
cluster 22
cluster 30
cluster 37
cluster 35
cluster 27
cluster 28
cluster 20
cluster 32
cluster 14
cluster 25
cluster 10
cluster 13
cluster 6
cluster 21
cluster 16
cluster 26
cluster 5
cluster 12
cluster 15
cluster 11
cluster 18
cluster 19
cluster 36
cluster 38
cluster 17
cluster 33

Figure S2 A. The similarity between tumor infiltrating T cell, L region infiltrating T cell and non-tumor region infiltrating T cell is compared by the overlap of tSNE plots. B. Cell percentage of each of the 40 clusters in T/L/N was plotted and compared. C. The cell percentage of CD4 Tem, CD8 Tem and Treg from different regions was compared. *P<0.05, **P<0.01, ***P<0.001. D. Comparison of cell percentage of certain T cell subtypes between different regions in HCC. Results are shown as mean ± SD, n=13. *P<0.05, **P<0.01, ***P<0.001, based on the student's t-test. E. Cell percentage of L-enriched T clusters in T/L/N of 13 enrolled patients. F. Heatmap showing the correlation among 40 T cell clusters. *P<0.05, **P<0.01, ***P<0.001.

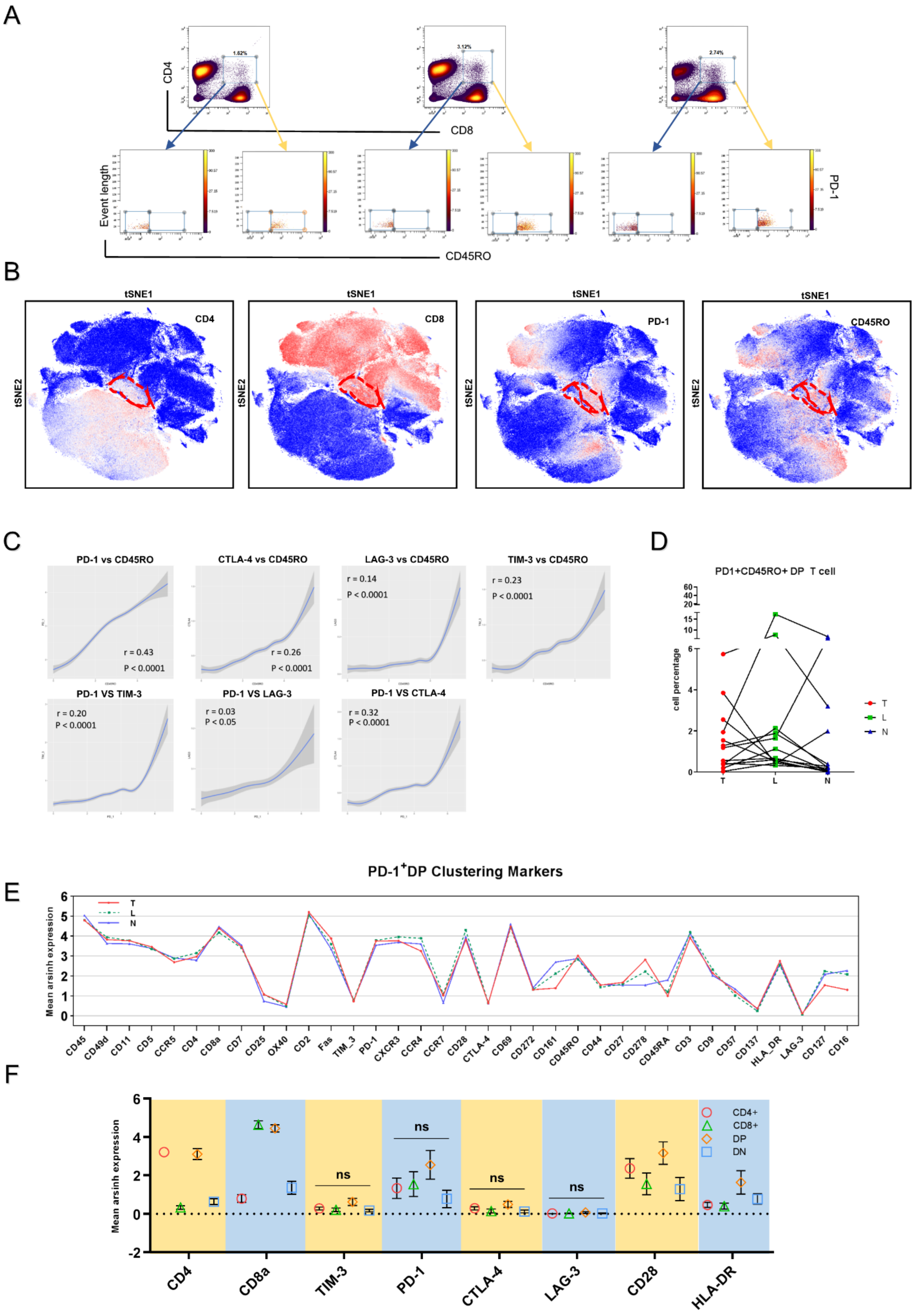
A
CD4
CD8
Event length
PD-1
CD45RO
1.62%
3.12%
2.74%
B
tSNE1
tSNE2
CD4
CD8
PD-1
CD45RO
C
PD-1 vs CD45RO
r = 0.43
P < 0.0001
CTLA-4 vs CD45RO
r = 0.26
P < 0.0001
LAG-3 vs CD45RO
r = 0.14
P < 0.0001
TIM-3 vs CD45RO
r = 0.23
P < 0.0001
PD-1 VS TIM-3
r = 0.20
P < 0.0001
PD-1 VS LAG-3
r = 0.03
P < 0.05
PD-1 VS CTLA-4
r = 0.32
P < 0.0001
D
PD1+CD45RO+ DP T cell
cell percentage
T
L
N
E
PD-1+DP Clustering Markers
Mean arsinh expression
CD45
CD49d
CD11
CD5
CCR5
CD4
CD8a
CD7
CD25
OX40
CD2
Fas
TIM_3
PD-1
CXCR3
CCR4
CCR7
CD28
CTLA-4
CD69
CD272
CD161
CD45RO
CD44
CD27
CD278
CD45RA
CD3
CD9
CD57
CD137
HLA_DR
LAG-3
CD127
CD16
F
Mean arsinh expression
ns
CD4+
CD8+
DP
DN
CD4
CD8a
TIM-3
PD-1
CTLA-4
LAG-3
CD28
HLA-DR

Figure S3 A. Flow plots show cells gated as DPT cells. DPT cells are then divided into 2 groups based on the expression level of CD45RO. Levels of CD454RO are shown for each of these populations. The bar chart depicts the expression intensity of PD-1. B. The expression level of CD4, CD8, PD-1 and CD45RO in 2D tSNE of total T cell pool. DPT cells are circled with a red line. DP CD45RO+/DP CD45RO- cells are separated with a red line. C. The correlations of the expression level of multiple immune checkpoint molecules were plotted. PD-1 vs CD45RO ($r=0.43$, $P<0.0001$). CTLA-4 vs CD45RO ($r=0.26$, $P<0.0001$). LAG-3 vs CD45RO ($r=0.14$, $P<0.0001$). TIM-3 vs CD45RO ($r=0.23$, $P<0.0001$). PD-1 vs TIM-3 ($r=0.20$, $P<0.0001$). PD-1 vs LAG-3 ($r=0.03$, $P<0.05$). PD-1 vs CTLA-4 ($r=0.32$, $P<0.0001$). D. Cell percentage of DP CD45RO+ PD-1+ T cells in total T cells from TIL/ LIL/ NIL of all 13 patients. E. Average mean expression levels of each of the 3 different regions in HCC were shown for the 35 clustering proteins in PD-1^{+}DPT cell. F. In L region of HCC, the comparison of the expression level of several clustering markers and immune checkpoint molecules was made among single positive/ DP CD45RO+/DP CD45RO-/ double negative T cells. Results are shown as mean ± SD, n=13. *P<0.05, **P<0.01, ***P<0.001, based on the student's t-test.

A

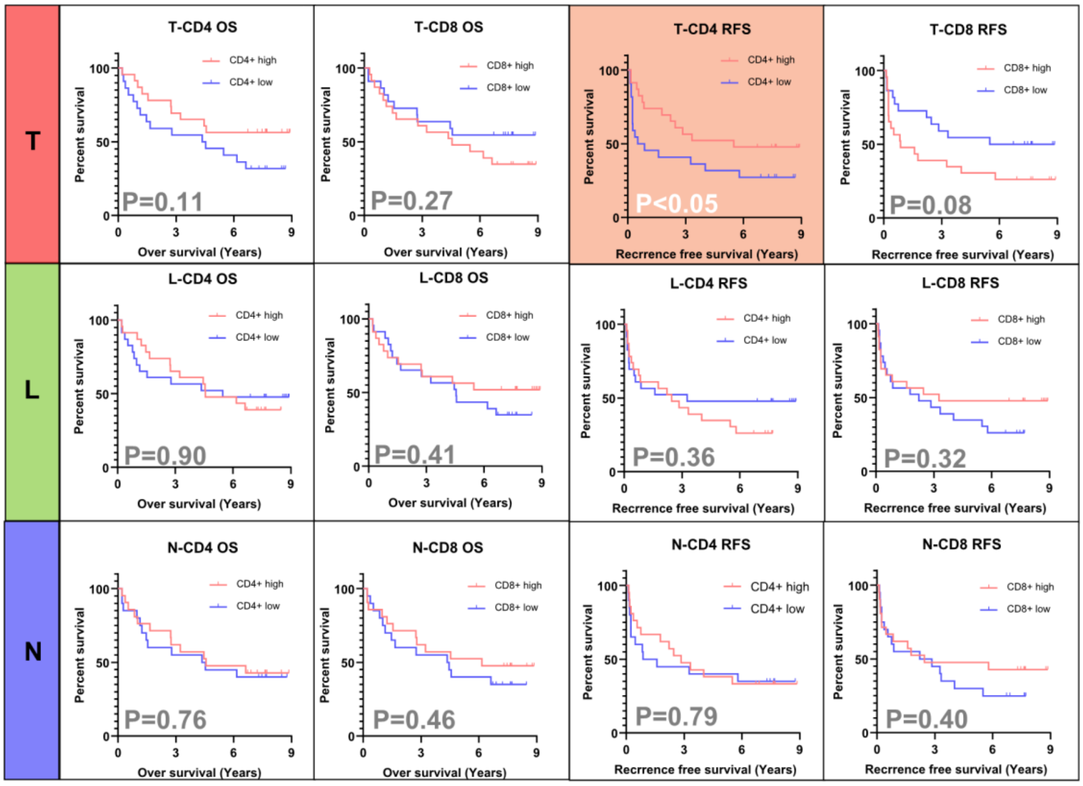

T
L
N
T-CD4 OS
T-CD8 OS
T-CD4 RFS
T-CD8 RFS
L-CD4 OS
L-CD8 OS
L-CD4 RFS
L-CD8 RFS
N-CD4 OS
N-CD8 OS
N-CD4 RFS
N-CD8 RFS
CD4+ high
CD4+ low
CD8+ high
CD8+ low
Percent survival
Over survival (Years)
Recrrence free survival (Years)
P=0.11
P=0.27
P<0.05
P=0.08
P=0.90
P=0.41
P=0.36
P=0.32
P=0.76
P=0.46
P=0.79
P=0.40

B

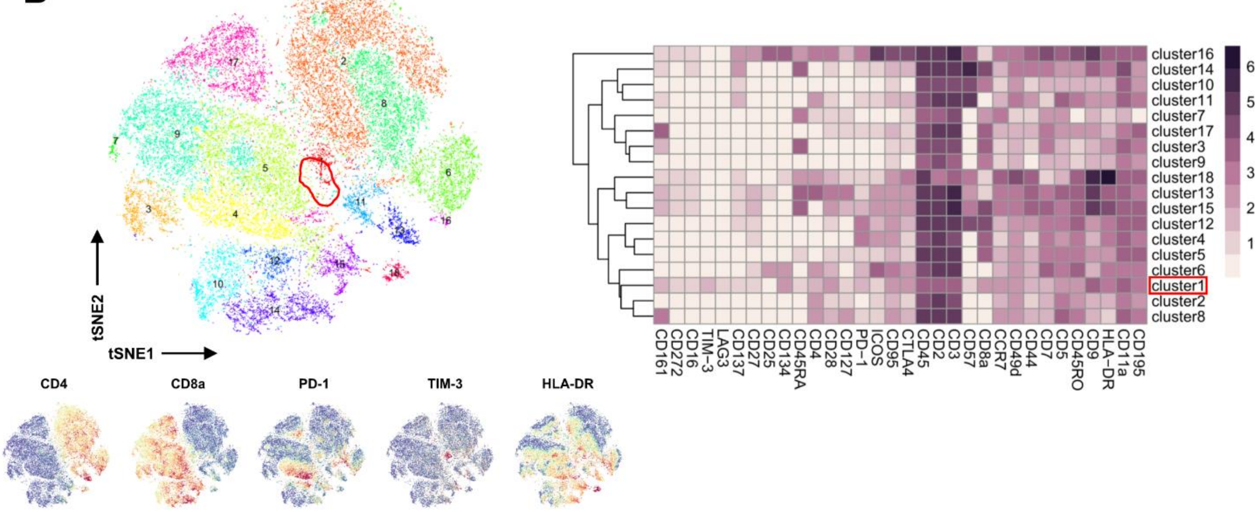
tSNE2
tSNE1
CD4
CD8a
PD-1
TIM-3
HLA-DR
cluster16
cluster14
cluster10
cluster11
cluster7
cluster17
cluster3
cluster9
cluster18
cluster13
cluster15
cluster12
cluster4
cluster5
cluster6
cluster1
cluster2
cluster8
CD161
CD272
CD16
TIM-3
LAG3
CD137
CD27
CD25
CD134
CD45RA
CD4
CD28
CD127
PD-1
ICOS
CD95
CTLA4
CD45
CD2
CD3
CD57
CD8a
CCR7
CD49d
CD44
CD7
CD5
CD45RO
CD9
HLA-DR
CD11a
CD195
6
5
4
3
2
1

C

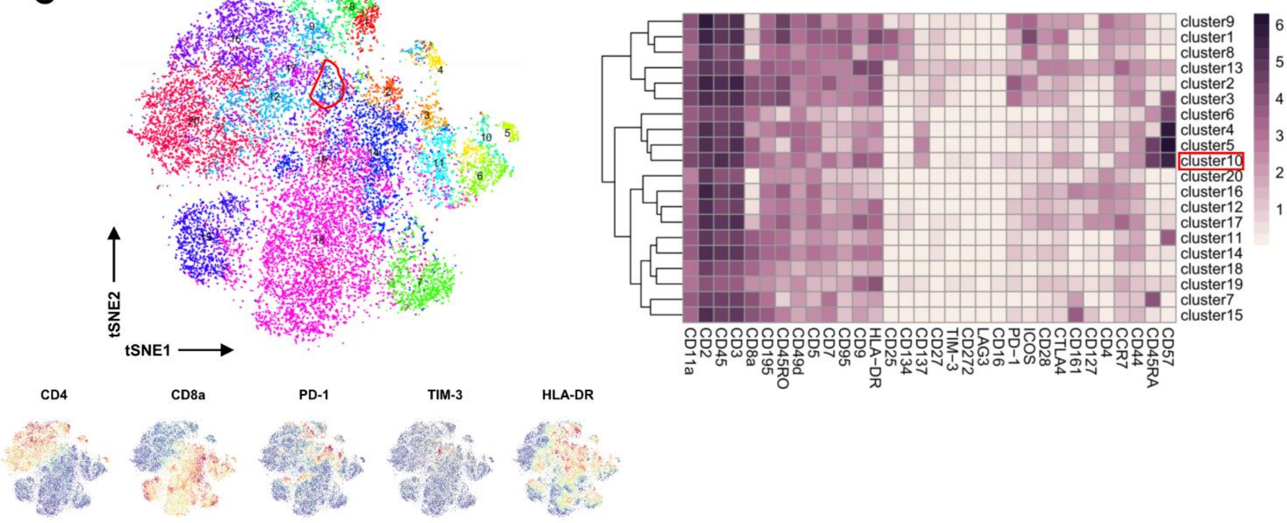
tSNE2
tSNE1
CD4
CD8a
PD-1
TIM-3
HLA-DR
cluster9
cluster1
cluster8
cluster13
cluster2
cluster3
cluster6
cluster4
cluster5
cluster10
cluster20
cluster16
cluster12
cluster17
cluster11
cluster14
cluster18
cluster19
cluster7
cluster15
CD11a
CD2
CD45
CD3
CD8a
CD195
CD45RO
CD49d
CD5
CD7
CD95
CD9
HLA-DR
CD25
CD134
CD137
CD27
TIM-3
CD272
LAG3
CD16
PD-1
ICOS
CD28
CTLA4
CD161
CD127
CD4
CCR7
CD44
CD45RA
CD57
6
5
4
3
2
1

D

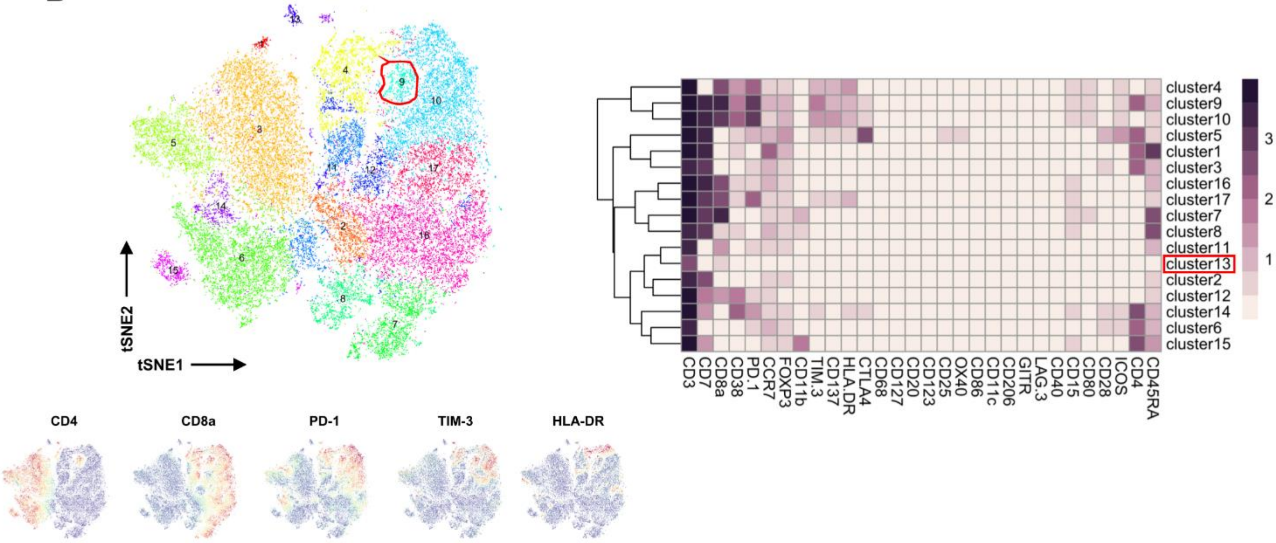
tSNE2
tSNE1
CD4
CD8a
PD-1
TIM-3
HLA-DR
cluster4
cluster9
cluster10
cluster5
cluster1
cluster3
cluster16
cluster17
cluster7
cluster8
cluster11
cluster13
cluster2
cluster12
cluster14
cluster6
cluster15
CD3
CD7
CD8a
CD38
PD.1
CCR7
FOXP3
CD11b
TIM.3
CD137
HLA.DR
CTLA4
CD68
CD127
CD20
CD123
CD25
OX40
CD86
CD11c
CD206
GITR
LAG.3
CD40
CD15
CD80
CD28
ICOS
CD4
CD45RA
3
2
1

Figure S4 A. Kaplan-Meier analysis of the correlation between CD4/CD8 single positive cell levels and overall survival (OS)/ recurrence-free survival (RFS). B. HCC cohort (30 cases): tSNE plots was drawn from a random sampling of 500 cells from each case. Phenograph clustering method was applied (left). Heatmap showed the expression feature of T cell clusters. C. ICC cohort (10 cases): tSNE plots was drawn from a random sampling of 500 cells from each case. Phenograph clustering method was applied (left). Heatmap showed the expression feature of T cell clusters. D. RCC cohort (70 cases): tSNE plots was drawn from a random sampling of 500 cells from each case. Phonograph clustering method was applied (left). Heatmap showed the expression feature of T cell clusters.

A

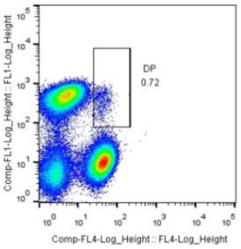

Figure S5 A. The gating information for DPT cells prepared for Single-cell sequencing.

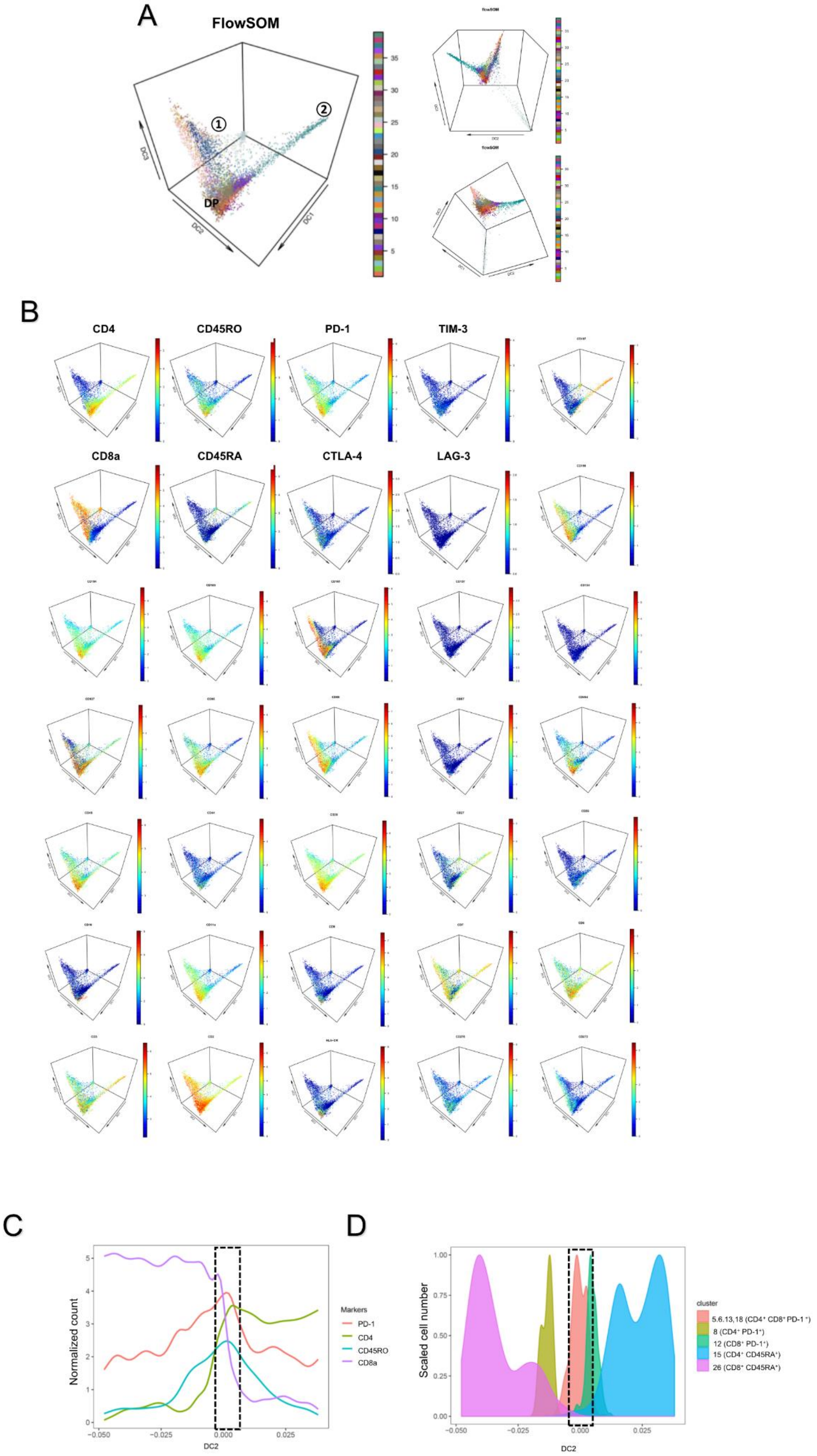
A
FlowSOM
①
②
DP
DC1
DC2
DC3
B
CD4
CD45RO
PD-1
TIM-3
CD8a
CD45RA
CTLA-4
LAG-3
C
Normalized count
DC2
Markers
PD-1
CD4
CD45RO
CD8a
D
Scaled cell number
DC2
cluster
5.6.13,18 (CD4⁺ CD8⁺ PD-1⁺)
8 (CD4⁺ PD-1⁺)
12 (CD8⁺ PD-1⁺)
15 (CD4⁺ CD45RA⁺)
26 (CD8⁺ CD45RA⁺)

Figure S6 A. Visualization of T cell clusters using first, second and third components of a diffusion map. Cells are colored by FlowSOM clusters. The two main branches are indicated with number 1 and 2. B. The expression patterns of 35 clustering makers and immune checkpoint molecules were shown in the diffusion map plots. C. Conditional mean expression of the indicated markers along diffusion component two. D. Histogram demonstrating the distribution of certain T cell clusters (DP PD-1$^+$, CD4$^+$ PD-1$^+$, CD8$^+$ PD-1$^+$, CD4$^+$ CD45RA$^+$, CD8$^+$ CD45RA$^+$) along diffusion component two.

Table S1. Chemical reagents used in CyTOF and analysis-related software/algorithm

Reagent name	Isotopes	Identifier	Source
**Antibodies (Human)**			
**CD45**	Y89	Cat# 3089003B	Fluidigm
**CD45RA**	169Tm	Cat# 3169008B	Fluidigm
**CD45RO**	165Ho	Cat# 3165011B	Fluidigm
**CD49d**	141Pr	Cat# 3141004B	Fluidigm
**CD5**	143Nd	Cat# 3143007B	Fluidigm
**CD57**	172Yb	Cat# 3172009B	Fluidigm
**CD69**	162Dy	Cat# 3162001B	Fluidigm
**CD7**	147Sm	Cat# 3147006B	Fluidigm
**CD8a**	146Nd	Cat# 3146001B	Fluidigm
**CD9**	171Yb	Cat# 3171009B	Fluidigm
**CD95**	152Sm	Cat# 3152017B	Fluidigm
**HLA-DR**	174Yb	Cat# 3174001B	Fluidigm
**Tim-3**	153Eu	Cat# 3153008B	Fluidigm
**CD278**	168Er	Cat# 3168024B	Fluidigm
**CD11a**	142Nd	Cat# 3142006B	Fluidigm
**CD127**	176Yb	Cat# 3176004B	Fluidigm
**CD134**	150Nd	Cat# 3150023B	Fluidigm
**CD137**	173Yb	Cat# 3173015B	Fluidigm
**CD152**	161Dy	Cat# 3161004B	Fluidigm
**CD16**	209Bi	Cat# 3209002B	Fluidigm
**CD161**	164Dy	Cat# 3164009B	Fluidigm
**CD183**	156Gd	Cat# 3156004B	Fluidigm
**CD194**	158Gd	Cat# 3158032A	Fluidigm
**CD195**	144Nd	Cat# 3144007A	Fluidigm
**CD197**	159Tb	Cat# 3159003A	Fluidigm
**CD2**	151Eu	Cat# 3151003B	Fluidigm
**CD223**	175Lu	Cat# 3175033B	Fluidigm
**CD25**	149Sm	Cat# 3149010B	Fluidigm
**CD27**	167Er	Cat# 3167002B	Fluidigm
**CD272**	163Dy	Cat# 3163009B	Fluidigm
**CD279**	155Gd	Cat# 3155009B	Fluidigm
**CD28**	160Gd	Cat# 3160003B	Fluidigm
**CD3**	170Er	Cat# 3170001B	Fluidigm
**CD4**	145Nd	Cat# 3140005B	Fluidigm
**CD44**	166Er	Cat# 3166001B	Fluidigm
**Antibodies (Mouse)**			
**CD8a**	168Er	Cat#3168003B	Fluidigm
**CD44**	150Nd	Cat#3150018B	Fluidigm
**CD197**	164Dy	Cat#3164013A	Fluidigm

**CD25**	151Eu	Cat#3151007B	Fluidigm
**CD69**	145Nd	Cat#3145005B	Fluidigm
**TIM-3**	162Dy	Cat#3162029B	Fluidigm
**CD16**	144Nd	Cat# 3144009B	Fluidigm
**PD-1**	159Tb	Cat# 3159006B	Fluidigm
**CD4**	172Yb	Cat# 3172003B	Fluidigm
**Chemicals, Peptides, and Recombinant Proteins**			
**Cisplatin**		Cat# 201192A	Fluidigm
**Iridium**		#201192A	Fluidigm
**Human TruStain FcX™**		#422302	Biolegend
**Software and Algorithms**			
**Cytobank**		https://support.cytobank.org/hc/en-us/articles/206336147-FCS-file-concatenation-tool	
**Concatenation tool**			Cytobank
**Cytofkit (tSNE, FlowSOM)**		https://bioconductor.org/packages/release/bioc/html/destiny.html	Chen et al. (1)

1. H. Chen, M. C. Lau, M. T. Wong, E. W. Newell, M. Poidinger, J. Chen, Cytofkit: A Bioconductor Package for an Integrated Mass Cytometry Data Analysis Pipeline. PLoS Comput Biol 12, e1005112 (2016).

Table S2. Clinical characteristics of the 46 HCC patients

patients ID	RFS day	Os day	pathology	differentiation	capsule	age	gender	AFP(500)	CEA(2.5)	CA19-9(37)	vascular invasion	cirrhosis	tumor thrombus	tumor size	tumor number	HBsAg	Total bilirubin (34)	Prothrombin time	Albumin	Ascites (ml)	Hepatic encephalopathy	Child-Pugh	BCLC	DPT cell percentage	DP PD-1+ cell percentage
**76739**	2971	2971	hcc	2	0	67	m	3.1	3.3	923.8	0	1	0	4.5	1	1	11.5	12.3	44.2	-	-	A	A	0.197393	0.063315
**77013**	2957	2957	hcc	3	0	71	m	4.7	3.8	90.6	0	0	0	7.5	1	0	12.6	12.2	41.8	-	-	A	A	0.039728	0.004957
**77159**	37	69	hcc	2	0	55	m			1000	0	1	1	5.5	2	1	37.6	11.5	38.9	<200	-	B	C	0.133634	0.018962
**77180**	190	285	hcc	2	1	52	m	546.1	2.4	90.6	0	1	1	4.5	2	1	6.3	13.6	42.7	-	-	A	C	0.192095	0.060279
**77390**	820	910	hcc	3	1	61	m	13.4	1.4	9.8	0	1	0	9	2	1	8.8	12.3	45.1	200	-	A	B	0.059866	0.04215
**77490**	78	328	hcc	3	0	63	m	358			0	1	0	5.5	1	1	13.6	13.3	46.7	-	-	A	A	0.121892	0.087275
**78065**	2925	2925	hcc	3	0	50	m	2.1	2.4	90.6	0	1	0	4	1	1	19.6	10.7	49.5	-	-	A	A	0.068282	0.022059
**78315**	54	70	hcc	2	0	35	m	11	1.2	14.2	0	0	0	9	2	1	9.7	12.3	38.2	-	-	A	B	0.080046	0.030162
**78393**	153	413	hcc	3	0	42	m	329.1	1.6	7.1	0	1	0	6	1	1	15.3	13.1	43.8	-	-	A	A	0.04119	0.018879
**78553**	170	261	hcc	3	0	47	m	141.3	4.3	9.3	0	0	0	9	2	1	9.6	12.6	38.9	<200	-	A	B	0.135734	0.041551

**78587**	42	122	hcc	2	0	61	f					1	1	10	1	0	12.6	12.3	27.5	<200	-	B	C	0.088131	0.045092
**78666**	59	2828	hcc	2	0	38	f	15.2	1.8	16.2	0	0	0	5	1	1	13.3	12.5	34.7	-	-	A	A	0.09383	0.01928
**78823**	1088	1452	hcc	2	0	77	m	1.3	2.6		0	1	0	6	2	0	9.4	11.5	41.4	-	-	A	B	0.188537	0.076345
**78861**	2899	2899	hcc	3	0	53	m	169.3	2.9	14.8	0	0	0	11	1	1	9.5	12	37.1	-	-	A	A	0.126741	0.025766
**79032**	1341	2210	hcc		0	73	m	3.7	3.7	12.1	0	0	0	7	1	0	20.9	11.1	42.7	-	-	A	A	0	0
**79308**	85	1824	hcc	2	0	45	m	95.8	1.8	36.8	0	0	0	5.5	2	1	15.3	12.4	38.5	-	-	A	B	0.147541	0.036988
**79830**	589	1077	hcc	3	0	52	m	5.2	4.7	272.4	1	1	0	6	1	1	13.3	11.3	40.5	-	-	A	C	0.057441	0.002611
**79957**	250	340	hcc	3	0	50	m	7	1	6.6	0	0	1	2	1	1	16	11	43.9	-	-	A	C	0.028369	0.007092
**79974**	947	1524	hcc	3	0	76	m	2.3	4.5	6.7	0	0	0	4	1	0	14.7	12.7	46.3	-	-	A	A	0.021955	0.001568
**80095**	2863	2863	hcc		0	64	m	6.8	2.4	7.7	0	0	0	8.5	1	0	12.5	10.1	41.5	-	-	A	A	0.261658	0.070596
**81072**	277	2834	hcc	3	0	45	m	87881	0.7		0	0	0	13.5	1	1	21.8	12	40.8	-	-	A	A	0.108747	0.053783
**89093**	80	177	hcc	3	0	54	m	7259	1.7	7.2	0	0	0	14	1	0	9.3	11	42.5	-	-	A	A	0.049254	0.049254
**89284**	2571	2571	hcc	3	0	68	m	4.2	8	0.6	0	0	1	5	2	0	240.9	11.6	41.8	-	-	B	C	0.199472	0.152576

**89352**	2574	2574	hcc	3	0	71	m	0	0		0	0	0	5	1	0	15.7	11.6	42.3	-	-	A	A	0.173865	0.130676
**89581**	42	72	hcc	3	0	42	m	1210			1	1	1	8.5	1	1	41.2	11.1	38.5	<200	-	B	C	0.172693	0.046595
**89626**	2568	2568	hcc	3	0	50	m	1210	0.6	58.8	0	1	0	8	1	1	10.7	12.1	38.9	-	-	A	A	0.204744	0.156471
**89802**	2557	2557	hcc	3	0	51	m	176.7	0.8	21.8	0	1	0	11	1	1	9.3	11.7	45.6	-	-	A	A	0.280744	0.177747
**89826**	2488	2488	hcc	3	0	49	m	70	1.8	23.9	0	0	0	11	1	0	10	12.5	39.2	-	-	A	A	0.239617	0.159744
**89863**	123	551	hcc	3	0	43	m	1210			1	1	0	6	2	1	6.8	11.3	40.6	-	-	A	C	0.099034	0.068438
**89990**	58	89	hcc	3	0	29	m				0	0	0	3.5	1	1	15.5	13.8	39.9	-	-	A	A	0.155729	0.107898
**90074**	2554	2554	hcc	3	0	38	f	1210			0	0	0	8	1	0	14.9	10.8	43.5	-	-	A	A	0.159166	0.071899
**90125**	2555	2555	hcc	3	0	44	m	8.6	6.4	44.6	1	1	0	6.5	1	1	14.1	12.3	48.5	-	-	A	C	0.15873	0.126984
**90265**	1111	1484	hcc	2	0	66	m	3.5	5.8	46.3	0	0	0	6.5	1	0	7.8	10.1	45.9	-	-	A	A	0.110708	0.001815
**90301**	2548	2548	hcc	3	0	54	m	917.5		15.2	1	1	0	5.5	1	1	21.8	12.3	45.7	-	-	A	C	0.195074	0.160591
**90336**	84	1514	hcc	3	0	61	m	1210			0	1	0	5.5	1	0	11.7	11.9	42.5	-	-	A	A	0.110475	0.086743
**90403**	2541	2541	hcc	3	0	56	m	9.1	1.2	17.3	1	1	0	9	1	1	15.5	11.3	43.1	-	-	A	C	0.212722	0.097595

**90494**	291	382	hcc	3	0	57	m	1210			1	1	1	8	1	1	28.3	12.1	31.8	-	-	A	C	0.120869	0.112236
**94815**	2436	2436	hcc	3	0	43	m	0			0	1	0	8	1	1	14.6	12.5	45.2	-	-	A	A	0.172391	0.073316
**95094**	56	513	hcc	3	0	65	m	2.2	1.9	14.6	0	0	0	5.5	1	1	7.3	10.8	34	-	-	A	A	0.103837	0.081264
**95612**	1832	2409	hcc	3	0	59	m	6.2	3.5	40.9	0	0	0	3	1	0	31.9	12.1	44.1	-	-	A	A	0.121287	0.071782
**99930**	2298	2298	hcc	3	0	52	m	2.9			0	1	0	8	1	1	8	12.5	39.5	-	-	A	A	0.237223	0.230473
**99998**	530	926	hcc		0	51	m	1210	1.4	9.1	0	0	0	16	1	0	24.5	11.5	37.7	-	-	A	A	0.021186	0.006356
**117829**	741	917	hcc		0	81	m	28.8			0	0	0	5	1	0	9.9	11.2	38.8	-	-	A	A	0.016704	0.011136
**118230**	2239	2239	hcc	3	0	40	m	0	0	0	0	1	0	2.5	1	1	17.2	13	41.8	-	-	A	A	0.147092	0.04656
**118292**	80	488	hcc	3	0	66	m	18.9	3.6	19.9	0	0	0	12	1	1	14.9	11.3	45.2	-	-	A	A	0.03838	0.007463
**118361**	1932	2055	hcc		0	66	m	6			0	1	1	4	1	0	19.3	12.7	32	-	-	A	C	0.077299	0.052533

Table S3. Clinical characteristics of the 13 HCC patients

No	Patient ID	Viral status	Grades	Sex	Race	Age	Tumor size(cm)	Tumor multiplicity	AFP level(ng/ml)	Pre treatment
**1**	207587	Hep B	III	M	China	52	4.5	2	3.8	Nil
**2**	207560	Hep B	III	M	China	57	9	1	16.1	Nil
**3**	209020	Hep B	I	F	China	75	3.9	1	1281	Nil
**4**	209270	Hep B	III	M	China	46	4.6	1	4.8	Nil
**5**	209283	Hep B	III	M	China	54	6.4	1	69.18	Nil
**6**	205596	N	IV	M	China	55	9.9	2	11199	Nil
**7**	205488	Hep B	III	M	China	50	7.3	1	3.6	Nil
**8**	205516	N	III	M	China	69	3	1	169	Nil
**9**	205832	N	III	M	China	66	7.2	1	9.9	Nil
**10**	205854	Hep B	III	F	China	65	5.5	1	34.2	Nil
**11**	205866	Hep B	III	F	China	71	3.2	1	9.9	Nil
**12**	206112	Hep B	III	M	China	61	4	1	1210	Nil
**13**	206169	Hep B	III	M	China	53	6.6	2	2.7	Nil

Table S4. Primary antibodies used in multiple immunohistochemistry

Species	Antigen	Source	Clone	Supplier	Application	Dilution
**Human**	CD4	Rabbit	EPR6855	abcam	WB, IHC-P, Flow Cyt, ICC/IF	1:80
**Human**	CD8	Rabbit		abcam	ICC/IF, WB, IHC-P, IHC-Fr	1:50
**Human**	PD-1	Mouse	MX033	MXB	IHC	1:100

Table S5. Primers used for real-time PCR

Primer names （human）	Sequences	
	Forward (5'~3')	Reverse (5'~3')
18S	CGGCTACCACATCCAAGGAA	GCTGGAATTACCGCGGCT
**XCL1**	TGCTCTCTCACTGCATACATTG	TGGTGTAGGTCTTGATTCTGCT
FOS	CCGGGGATAGCCTCTCTTACT	CCAGGTCCGTGCAGAAGTC
CRTAM	GACGCTCACTCTAAAGTGTGTC	CTTGCAGGGTTACGTTAGGCA
CCL4	CTGTGCTGATCCCAGTGAATC	TCAGTTCAGTTCCAGGTCATACA
GZMB	CCCTGGGAAAACACTCACACA	GCACAACTCAATGGTACTGTCG
AHI1	ATTGAGGAACACACAGTTAGCAA	GGCACCGTCTTTATCACCTTTT
GZMH	CTGGCTGGGGTTATGTCTCAA	GGCTACGTCCTTACACACGAG
CHST12	CTTCTACTTGCACACGTCCTT	CTCCGTCTCCTTTCTGGGAA
TNFRSF9	AGCTGTTACAACATAGTAGCCAC	GGACAGGGACTGCAAATCTGAT
PDCD1	CCAGGATGGTTCTTAGACTCCC	TTTAGCACGAAGCTCTCCGAT
KLRD1	CAGGACCCAACATAGAACTCCA	GGAAATGAAGTAACAGTTGCACC
TUBA4A	TGAGATCCGAAATGGCCCATA	TAGTGACCACGGGCATAGTTG
HLA-DRB1	GAGCAGGTTAAACATGAGTGTCA	CTCTCCACAACCCCGTAGT
CCL3	AGTTCTCTGCATCACTTGCTG	CGGCTTCGCTTGGTTAGGAA
HLA-DQA1	TCGCTCTGACCACCGTGAT	AGGGACCGTAAAACTGGTACAA
CST7	GTGTGAAGCCAGGATTTCCTAA	TGTCGTTCGTGCAGTTGTTGA
SLC7A5	CCGTGAACTGCTACAGCGT	CTTCCCGATCTGGACGAAGC
DUSP4	GGCGGCTATGAGAGGTTTTCC	TGGTCGTGTAGTGGGGTCC
ITM2A	ATCCTGCAAATTCCCTTCGTG	CAGGTAAGCAGTCATTCCCTTT

Table S6. Fluorochrome-conjugated antibodies used in flow cytometry

Species	Source	Antigen	Fluorochrome	Clone	Supplier
**Human**	Mouse	CD45	APC-Cy™7	2D1	BD Bioscience
**Human**	Mouse	CD3	Alexafluor700	500A2	BD Bioscience
**Human**	Mouse	CD8	BB515	RPA-T8	BD Bioscience
**Human**	Mouse	CD45RO	APC	UCHL1	BD Bioscience
**Human**	Mouse	IFN-γ	PE	4S.B3	BD Bioscience
**Human**	Mouse	TNFα	BV650	MAb11	BD Bioscience
**Human**	Mouse	IL-2	BV421	5344.111	BD Bioscience
**Human**	Mouse	Fosp3	PE-CF594	259D/C7	BD Bioscience
